# Unusual effects of a nanoporous gold substrate on cell adhesion and differentiation because of independent multi-branch signaling of focal adhesions

**DOI:** 10.1007/s10856-023-06760-0

**Published:** 2023-10-26

**Authors:** Peizheng Wu, Kazuya Yanagi, Kazuki Yokota, Masataka Hakamada, Mamoru Mabuchi

**Affiliations:** Graduate School of Energy Science, Kyoto University, Yoshidahonmachi, Sakyo, Kyoto 606-8501 Japan

**Keywords:** Nanoporous gold, Surface topology, Focal adhesion, Cell adhesion, Differentiation

## Abstract

A variety of cell behaviors, such as cell adhesion, motility, and fate, can be controlled by substrate characteristics such as surface topology and chemistry. In particular, the surface topology of substrates strongly affects cell behaviors, and the topological spacing is a critical factor in inducing cell responses. Various works have demonstrated that cell adhesion was enhanced with decreasing topological spacing although differentiation progressed slowly. However, there are exceptions, and thus, correlations between topological spacing and cell responses are still debated. We show that a nanoporous gold substrate affected cell adhesion while it neither affected osteogenic nor adipogenic differentiation. In addition, the cell adhesion was reduced with decreasing pore size. These do not agree with previous findings. A focal adhesion (FA) is an aggregate of modules comprising specific proteins such as FA kinase, talin, and vinculin. Therefore, it is suggested that because various extracellular signals can be independently branched off from the FA modules, the unusual effects of nanoporous gold substrates are related to the multi-branching of FAs.

**Graphical Abstract:**

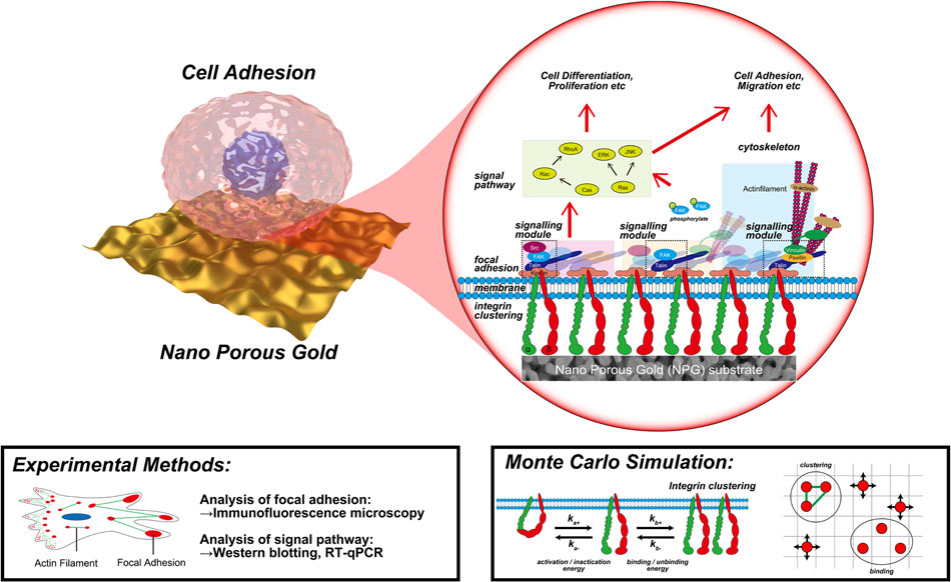

## Introduction

Substrates affect a variety of cell behaviors, such cell adhesion, migration, proliferation, and differentiation, via the surface topology [1–18], surface chemistry [19–21], and rigidity of the substrates [22, 23]. Topological spacing is a critical factor affecting cell behavior [1–18]. Various studies have shown that the cell adhesion was strengthened with decreasing topological spacing, while the cell differentiation progressed slowly with decreasing spacing [8–14]; specifically, the surface topology affected both the cell adhesion and fate, and the tendency of cell adhesion with respect to the topological spacing was the opposite of that of differentiation. The threshold topological spacing was approximately 70 nm, which corresponds to the molecular size of talin or α-actinin; at larger spacings, the cell adhesion was rapidly reduced [8, 10, 14]. The spacing trend was also demonstrated using Monte Carlo simulations [24]. However, Lee et al. [15] showed that a decrease in spacing upregulated the proliferation, resulting in enhanced differentiation. Thus, the correlations between topological spacing and cell behaviors are still debated.

Integrins are transmembrane proteins through which extracellular signals are transduced inside a cell, and they play a vital role in the transmission of signals related to the surface topology. The allosterically induced conformational changes of integrin are complex [25, 26], and they include a bent/extended conformational change and a closed/open conformational change. In addition, a focal adhesion (FA) is formed by the activation of an integrin with the extended and open structure [27]. FA comprises various proteins such as scaffold types and adaptor types. For example, focal adhesion kinase (FAK) plays a central role in recruitment of proteins comprising FA, and talin and vinculin play a critical role in the catch bond of integrin whose lifetime increases with applied stress [28, 29]. The large macromolecular assembly induces complex effects involving integrins.

The effects of nanoporous gold (NPG) have been investigated on cell behavior [30–41]. The surface of NPG is hyperpolarized because of a local large compressive strain, which leads to specific effects of NPG [39]. The intensity of hyperpolarization at the NPG surface depends on the pore size [40]. The motivation of the present work is to understand the effects of NPG substrates with different pore sizes on cell adhesion and differentiation. The conformation of integrin is allosterically changed by binding to ligands [25, 26]. Therefore, denaturation of extracellular matrix (ECM) induced by substrates leads to abnormal changes in the integrin conformation, which affects cell behavior. However, NPG causes little change in collagen conformation, while it reduces the cell adhesion [38]. Thus, NPG substrates are suitable for the investigation of the effects of surface topology without abnormal changes in the integrin conformation. As a result, this work demonstrates that NPG has unusual effects on cell adhesion and differentiation that are contrary to the previous findings.

## Materials and methods

### Experimental procedures

#### Reagents and supplies

Dulbecco’s modified Eagle medium (DMEM) (1.0 g/L glucose), Dulbecco’s phosphate-buffered saline (DPBS), and trypsin-ethylenediaminetetraacetic acid (EDTA) solution were purchased from Nacalai Tesque (Kyoto, Japan). Mesenchymal Stem Cell Growth Medium Bullet Kit™ (PT-3001), hMSC Osteogenic Differentiation Medium Bullet Kit™ (PT-3002), and hMSC Adipogenic Differentiation Medium Bullet Kit™ (PT-3004) were purchased from Lonza (Walkersville, MD, USA). Fetal bovine serum (FBS) and antibiotic-antimycotic solution 100× were purchased from Gibco (Waltham, MA, USA).

#### Cell culture and maintenance

Human embryo fibroblasts (HFs) (OUMS-36T-1) were purchased from JCRB Cell Bank (Osaka, Japan) and cultured in a 37 °C and 5% CO_2_ incubator with DMEM (Nacalai Tesque) supplemented with 10% FBS (Gibco) and 5% antibiotic-antimycotic solution 100× (Gibco). The medium was changed every two days. HFs were passaged when they became sub-confluent (approximately 70% confluent).

Normal bone marrow-derived human mesenchymal stem cells (hMSCs) were purchased from Lonza and cultured in a 37 °C and 5% CO_2_ incubator with hMSC basal medium containing SingleQuots™ Supplements and Growth Factors (PT-3001, Lonza). The medium was changed every two days. HFs were passaged when were sub-confluent (approximately 80% confluent). hMSCs (passage 3–5) were used in differentiation experiments.

#### Preparation of the nanoporous gold substrate

An NPG substrate was fabricated by radio frequency (RF) sputtering and dealloying (chemical corrosion of silver by nitric acid) as follows: first, a 1000-nm thick pure gold film (>99.9 mass%) was sputtered on a Φ = 22 mm micro cover glass (Matsunami Glass Ind., Ltd., Osaka, Japan) with a RF sputtering apparatus SVC-700RF (Sanyu Electron Co., Ltd., Tokyo, Japan). Next, a 300-nm thick, gold–silver alloy (atom ratio of gold:silver = 3:7) was sputtered on the gold thin film. The alloy was then immersed in nitric acid whose concentration was 70% and washed with DPBS (Nacalai Tesque) to fabricate an NPG substrate. Two ways were employed to change the pore sizes of the NPG substrates: one was to change the conditions of dealloying and the other was to change the conditions of heat treatment after dealloying (Table S1), because it was difficult to control the pore sizes with only one way. The nanostructures of the NPG substrates were observed using a scanning electron microscope (SEM, SU-6600, Hitachi High Technologies, Tokyo, Japan). The quantitative chemical composition of the NPG substrates was analyzed with energy dispersive X-ray spectroscopy (EDXS). Five areas from three different NPG samples were randomly selected to quantify the pore size and ligament size using ImageJ software.

#### Adhesion rate of HFs on NPG

HFs were trypsinized from a culture dish, then seeded on flat gold (FG) and NPG substrates at a density of 5.0 × 10^3^ cells/cm^2^. After 4-, 24-, and 48-hour cultures in DMEM (Nacalai Tesque) in a 37 °C and 5% CO_2_ incubator, the culture medium was removed to quantify the cell adhesion rates and circularities. Cells on the substrates were fixed with a 4% paraformaldehyde phosphate buffer solution (PFA, Nacalai Tesque) for 10 min, permeabilized with 0.5% Triton X-100 (Nacalai Tesque) in DPBS for 10 min, and then blocked with Blocking One Histo (Nacalai Tesque) for 30 min. To visualize the cytoskeleton and nuclei, all cell samples were immunofluorescence stained by Acti-stain™ 555 Fluorescent Phalloidin (Cytoskeleton, Inc., Denver, CA, USA) and 4’,6-diamidino-2-phenylindole (DAPI, Sigma Aldrich, Burlington, MA, USA), and observed using a fluorescence microscope BX-53 (Olympus, Tokyo, Japan). The number of HFs were measured by counting nuclei using ImageJ. The adhesion rate was defined as the ratio of the cell density after 4-, 24- and 48-hour culture to seeding density. The number of HFs adhered to the FG was used as a standard, and the adhesion rates for NPG were normalized to the standard. The circularities of HFs adhered on the FG and NPG substrates were investigated with ImageJ to quantify the morphological character of the HFs. The circularity was given by 4π*A*/*L*^2^, where *A* is the area of a cell and *L* is the perimeter of a cell.

#### Immunofluorescent staining of focal adhesion and quantification

Immunofluorescent staining was performed to visualize FA proteins: vinculin, paxillin, and FAK. As mentioned in Section 2.1.4, cells on substrates were fixed with 4% PFA, permeabilized with 0.5% Triton X-100, and blocked with Blocking One Histo. Substrates were then incubated with primary antibodies overnight at 4 °C: vinculin (ab129002, abcam, Cambridge, UK), paxillin (ab32084, abcam), and phospho Y397 FAK (ab81298, abcam) following dilution protocols before labeling with secondary antibodies: Goat Anti-Rabbit IgG H&L Alexa Fluor® 488 (ab150077, abcam) or Goat Anti-Rabbit IgG H&L Alexa Fluor® 647 (ab150083, abcam) for 30 min following dilution protocols. Actin filament and nuclei were stained for 30 min using Acti-stain™ 555 Fluorescent Phalloidin and DAPI, then substrates were imaged using a fluorescence microscope BX-53 with a UPLX Apo 10× objective lens or BZ-X710 microscope (Keyence, Osaka, Japan) with a Plan Apo Lambda 10× or 20× objective lens. Using a protocol designed to quantify the FAs [42, 43], the average size of the FAs, number of FAs in a cell adhesion site, cell adhesion site, and FA density were quantified using ImageJ software.

#### Western blotting (WB)

The expression levels of FA proteins were quantified using western blotting (WB) at room temperature (RT). Whole cell proteins were extracted using NuPAGE™ LDS Sample Buffer (Thermo Fisher, Waltham, MA, USA), loaded on Bolt™ Bis-Tris Protein Gel (Thermo Fisher), and blotted onto 0.45 μm PVDF membranes (Thermo Fisher). Proteins were quantified using a Qubit™ Protein Broad Range Assay and Qubit 4 Fluorometer (Thermo Fisher) following the manufacturer’s instruction. Membranes were finally incubated with primary antibodies: GAPDH (AM4300, Thermo Fisher), vinculin (ab129002, abcam), paxillin (ab32084, abcam), FAK (ab40794, abcam), and phospho Y397 FAK (ab81298, abcam) and secondary antibodies: Goat Anti-Rabbit IgG H&L HRP (ab205718, abcam) and Goat Anti-Mouse IgG H&L HRP (G21040, Thermo Fisher) using iBind Western System (Thermo Fisher). All membranes were imaged using a ChemiDoc Touch MP system (Bio-Rad Laboratories, Hercules, CA, USA). Bands were quantified using ImageJ, and statistical data were analyzed using GraphPad Prism 9.

#### Differentiation of hMSCs on NPG

hMSCs were cultured on the NPG substrates, and osteogenesis and adipogenesis were initiated to investigate the effects of NPG on the differentiation of hMSCs. For osteogenic differentiation, hMSCs were trypsinized, seeded on the NPG substrates at a density of 3.1 × 10^3^ cells/cm^2^, and pre-cultured for 24 h to make cells adhere to the NPG. hMSCs were then differentiated using the hMSC Osteogenic Differentiation Medium Bullet Kit™ (PT-3002) and the culture medium was changed every 3 d for 21 d. For adipogenic differentiation, hMSCs were trypsinized, seeded on the NPG substrates at a density of 2.1 × 10^4^ cells/cm^2^, and pre-cultured until near confluence. Then, three cycles of induction/maintenance were performed using the hMSC Adipogenic Differentiation Medium Bullet Kit™ (PT-3004) following instructions before maintenance for seven days. All of the processes for differentiation were performed according to the manufacturer’s instructions.

#### Differentiation evaluation

An osteogenic differentiation assay was performed using an Alkaline Phosphatase (ALP) Staining Kit (AK20, Cosmo Bio, Tokyo, Japan) and ALP Assay Kit (ab83369, abcam). For ALP staining, hMSCs cultured on the NPG substrates were washed three times with DPBS, fixed for 20 min, incubated in a chromogenic solution for 20 min, washed by distilled water to stop the reaction, and finally imaged with a BX53 microscope. For quantification of ALP, cell lysates were generated, homogenized, reacted with the ALP reaction solution for 60 min at room temperature under protection from light. The absorbance was then measured at 405 nm with a Spark® microplate reader (TECAN, Zurich, Swiss).

An adipogenic differentiation assay was performed by Oil Red O staining (01391, Sigma–Aldrich). hMSCs on the NPG substrates were fixed with 4% PFA for 30 min, washed with DPBS three times, incubated with 60% Oil Red solution for 20 min, washed with distilled water or 60% isopropanol three times, and finally imaged with a BX53 microscope. Oil Red O quantification was then performed. Stained Oil Red O was dissolved in 100% isopropanol and its absorbance was measured at 510 nm using a Spark® microplate reader (TECAN).

#### Real time quantitative polymerase chain reaction (RT-qPCR)

Genetic analyses were performed using RT-qPCR to quantify the expression levels of the differentiation marker of osteogenesis and adipogenesis. The total RNA was isolated using a PureLink™ RNA Mini Kit (Invitrogen, Waltham, MA, USA), and quantified by a Qubit™ RNA Board Range Assay Kit (Invitrogen) and Qubit 4 Fluorometer. cDNA was generated using a SuperScript™ IV VILO™ Master Mix (Invitrogen) with ezDNase™ Enzyme (Invitrogen). RT-qPCR was performed on a StepOnePlus™ Real Time PCR System (Applied Biosystems, Waltham, MA, USA) with a TaqMan™ Fast Advanced Master Mix (Applied Biosystems). The following TaqMan™ probes were used: glyceraldehyde 3-phosphate dehydrogenase (GAPDH, Hs02786624_g1), runt related transcription factor 2 (RUNX2, Hs01047973_m1), bone gamma-carboxyglutamate protein (BGLAP, Hs01587814_g1), peroxisome proliferator activated receptor gamma (PPARγ, Hs01115513_m1), fatty acid binding protein 4 (FABP4, Hs01086177_m1), and lipoprotein lipase (LPL, Hs00173425_m1). The expression levels of mRNA were normalized by GAPDH as an internal control.

#### Analysis and statistics

The data are presented as mean ± standard error of the mean (s.e.m). All statistical analyses are performed with Prism 9 (Graph Pad, San Diego, CA, USA). The *p*-values were analyzed with one-way analysis of variance (ANOVA) followed by a Tukey’s post-hoc test. Differences were considered significant at *p* < 0.05.

### Computational details

#### Modeling of NPG substrates

The structure of NPG is related to phase separation of the gold-silver alloy. We modeled the NPG substrate with a pore size of 20 nm by calculating the stable structure of two-phase separation using the Cahn–Hilliard equation [44]. We then modeled the NPG substrates with pore sizes of 50, 75, 100, and 150 nm by enlarging the 20 nm model.

#### Clustering of integrins

Clustering of integrins occurs by three reactions of the activation of a single integrin, the binding of a single integrin to ECM, and the association between integrins [24, 45, 46]. The integrin activation can be described by [24, 45, 46]

1 $$\frac{{k}_{a+}}{{k}_{a-}}=\exp \left(\frac{-{E}_{a}}{kT}\right)$$

where *k*_*a+*_ and *k*_*a−*_ are the reaction rates for activation and inactivation of a single integrin, *E*_*a*_ is the activation energy, *k* is the Boltzmann constant, and *T* is the absolute temperature. In the present work, the value of *E*_*a*_ was set to a constant (=3 *kT* [46]) because integrin activation is related to the inside-out signaling and the NPG has no effect on the integrin activation. The integrin activation and inactivation were calculated using Eq. (1), where *k*_*a+*_ was set to 10 s^*−*1^ [45].

The integrin binding can be given by [24, 45, 46]

2 $$\frac{{k}_{b+}}{{k}_{b-}}=\exp \left(\frac{{E}_{b}}{kT}\right)$$

where *k*_*b+*_ and *k*_*b−*_ are the reaction rates for binding and unbinding of a single integrin, and *E*_*b*_ is the binding energy. To consider the effects of NPG substrates on the integrin binding, the binding energy was given by

3 $${E}_{b}=\alpha c\frac{B}{{r}_{p}}+Ac(kT)$$

where *α* is the intensity of the NPG effect on the binding (normally = 1), *c* is the concentration of gold, *r*_*p*_ is the pore size of NPG substrate, and *A* and *B* are constants. In the present work, *A* and *B* were set to 3.5 and *−*12, respectively. The NPG substrate affected the binding of integrins to the ECM [38], and the effects of NPG were reduced with increasing pore size [40]. Additionally, the binding energy was in the range of 0–10 *kT* [24, 45]. These were considered to determine the values of *A* and *B*. The integrin binding and unbinding were calculated using Eq. (2), where *k*_*b+*_ was set to 10 s^*−*1^ [45]. The value of *α* was changed from 1 to 0.5 when NPG less affected the binding and association energies, and to 2.5 when NPG more affected the binding and association energies.

The integrin association can be given by [24, 45, 46]

4 $$\frac{{k}_{c+}}{{k}_{c-}}=\exp \left(\frac{{E}_{c}}{kT}\right)$$

where *k*_*c+*_ and *k*_*c−*_ are the reaction rates for the association and disassociation of integrins, and *E*_*c*_ is the association energy. Assuming that the association energy did not depend on the pore size of NPG, the value of *E*_*c*_ was set to a constant of 6.2 *kT*. Alternatively, assuming that the pore size affected the association energy, *E*_*c*_ was given by

5 $${E}_{c}=\beta c\frac{{r}_{p}}{{r}_{max}}+C(kT)$$

where *β* is the intensity of the NPG effect on the association (normally = 1), *r*_*max*_ is the pore size when the integrin clustering for an NPG substrate corresponds to the integrin clustering for a flat gold substrate, and *C* is a constant. The value of *C* was set to 5.2. The association energy was in the range of 0–10 *kT* [24, 45], which was considered to determine the value of *C*. The integrin clustering for the NPG substrate with a pore size of 150 nm corresponded to the one for a flat gold substrate, as shown later. Therefore, *r*_*max*_ was 150 nm. Additionally, the association energy for a flat gold substrate was the same as that for the NPG substrate with a pore size of 150 nm. The integrin association and disassociation were calculated using Eq. (4), where *k*_*c+*_ was set to 1 s^*−*1^ [45]. The value of *β* was changed from 1 to 0.5 when NPG less affected the binding and the association energies, and to 2 when NPG more affected the binding and association energies.

#### Monte Carlo simulation

A square 6 × 6 μm^2^ membrane was created, divided into 100 × 100 square grids with uniform sides, and then 1000 integrins were randomly placed on the patch for Monte Carlo (MC) simulations of integrin clustering. Integrins were allowed to randomly diffuse to neighboring grids at each calculation step in the cases of inactivated integrins and activated integrins without association. Periodic boundary conditions were imposed. Ligands of ECM were uniformly distributed on grids such that one ligand was present on one grid. Assignment simulations were performed where an integrin underwent either a reaction event (activation/binding/association) or diffusion according to a specified probability at each time step. The interval time of the MC step was 10 ms [46]. MC calculations were performed until an equilibrium state was reached. We took the area of FAs as the number of integrins in a cluster, and measured the average area of FAs by averaging the areas of the FA of 10% of the largest clusters.

## Results

### Fabrication of NPG substrates with different pore sizes

**Fig. 1:**
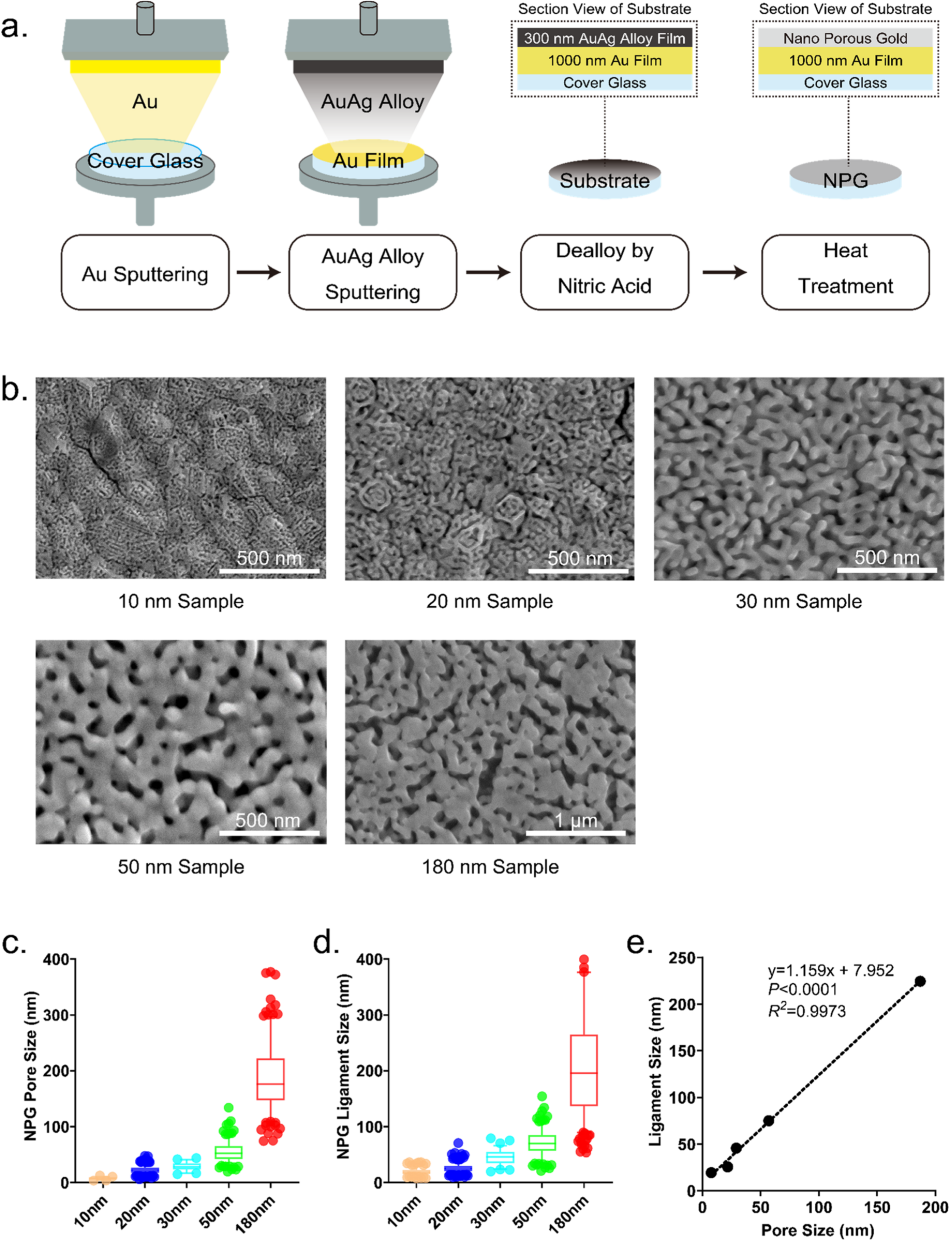
Fabrication and characterization of nanoporous gold (NPG) substrates. **a** Schematic illustration of the four step process for fabrication of an NPG substrate. Step 1: sputtering of Au on a cover glass; Step 2: sputtering of AuAg alloy on an Au film; Step 3: dealloying by nitric acid; Step 4: heat treatment. **b** Scanning electron microscope (SEM) images of NPG samples with the pore sizes of 10, 20, 30, 50, and 180 nm. **c** Quantitative analysis of the pore size (mean ± s.e.m, *N* > 50). The pore sizes were 7.649 ± 0.244, 23.060 ± 0.343, 29.175 ± 1.029, 54.411 ± 1.195 and 186.641 nm ± 3.583 nm for 10, 20, 30, 50, and 180 nm samples, respectively. **d** Quantitative analysis of ligament size (mean ± s.e.m, *N* > 50). The ligament sizes were 19.472 ± 0.436, 25.905 ± 0.395, 45.667 ± 1.533, 71.251 ± 1.527 and 210.937 nm ± 5.170 nm for 10, 20, 30, 50, and 180 nm samples, respectively. **e** Correlation between pore size and ligament size. *P* < 0.0001, *R*^*2*^ = 0.9973

NPG substrates were fabricated by dealloying, and heat treated to increase the pore size (Fig. 1a). The pore size was controlled to above and below a threshold spacing of 70 nm. In particular, it was controlled to investigate effects of pore size in the range below the threshold because the intensity of hyperpolarization strongly depends on the pore size in the small pore size range. As a result, the pore sizes were approximately 10, 20, 30, 50, and 180 nm (Fig. 1b–d). The pore size was closely correlated with the ligament size (Fig. 1e). Hence, the pore size is used as the parameter to describe the topographic features of the NPG substrate in the present work.

### Effects of pore size on the cell adhesion

**Fig. 2:**
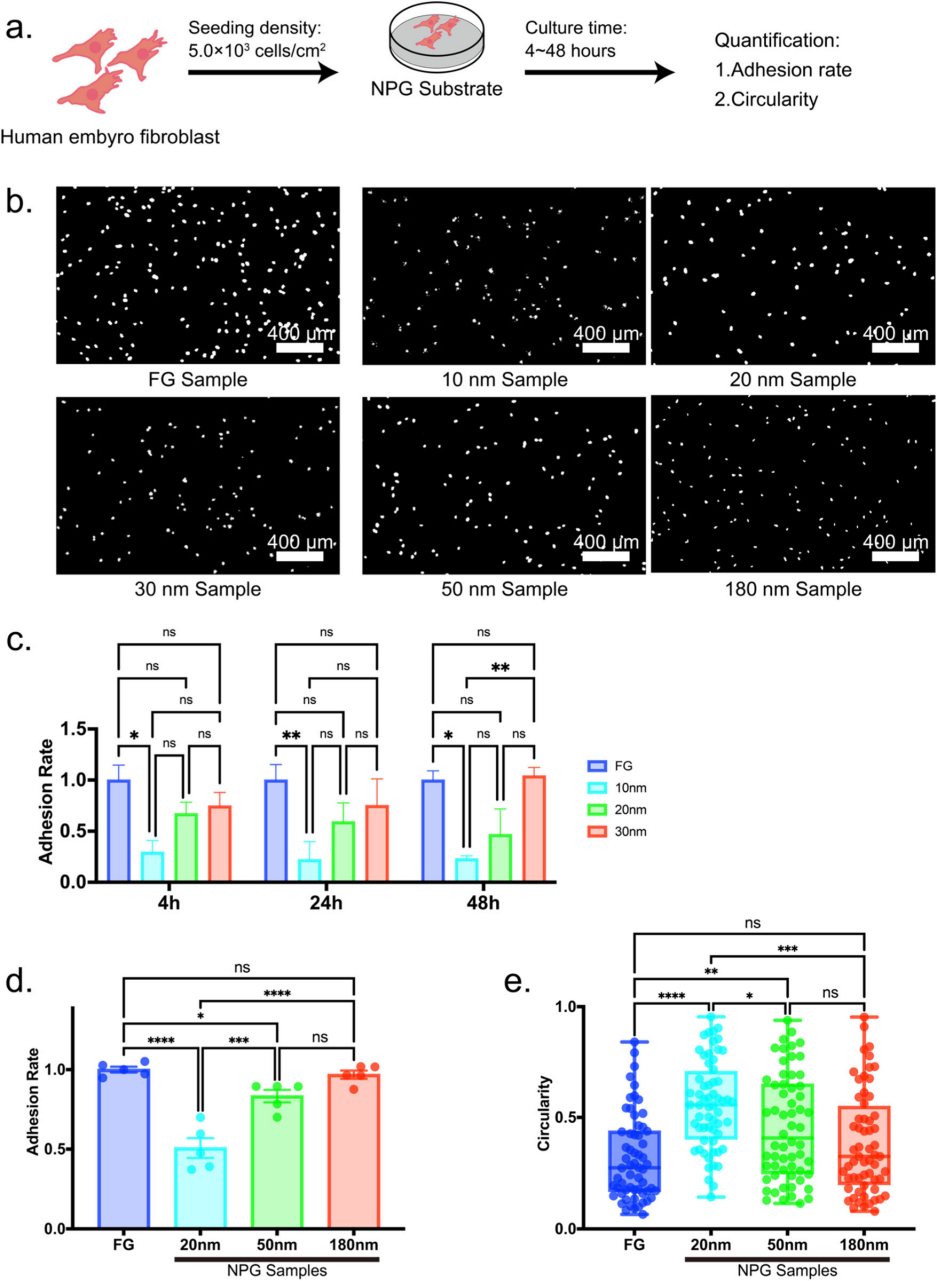
Cell adhesion of human embryo fibroblasts (HFs) on nanoporous gold (NPG) substrates. **a** Schematic illustration of the cell adhesion experiment. HFs were seeded to NPG substrates at a density of 5.0 × 10^3^ cells/cm^2^, cultured for 4, 24, and 48 h before quantification of the cell adhesion rate. **b** Immunofluorescent staining of nuclei (DAPI) for flat gold (FG), 10, 20, 30, 50, and 180 nm NPG samples. Images were binarized using ImageJ. **c** Variations in cell adhesion rate with time for FG, 10, 20, and 30 nm NPG samples. **d** Cell adhesion rate after 24 h culture for FG, 20, 50, and 180 nm NPG samples. The cell adhesion rate increased with increasing pore size (**c**, **d**). **e** Circularities of cells adhered on FG, 20, 50, and 180 nm NPG samples (*N* = 60). All data were presented as the mean ± s.e.m. The *P*-values were analyzed with one-way analysis of variance (ANOVA) followed by a Tukey’s post hoc test. *, **, ***, and **** indicate *P* < 0.05, *P* < 0.01, *P* < 0.001, and *P* < 0.0001; ns indicates not significant

The cell adhesion was reduced by the NPG substrate (Fig. 2b–d) although the results of cell adhesion had some variations in data. Tan et al. [35] previously demonstrated that the cell proliferation was reduced by the NPG substrate. Thus, the NPG affected the cell activity, potentially because of the hyperpolarization of the NPG surface [39]. Notably, the cell adhesion was reduced with decreasing pore size, which corresponds to previous NPG results [34]. The finding is contrary to previous results on other substrates with a surface topology where the cell adhesion was enhanced with decreasing topological spacing. Hence, the tendency of reduced adhesion with decreasing pore size is suggested to be specific to the NPG substrate.

The fact that the cell adhesion was reduced with decreasing pore size was independent of the culture time in the range investigated (Fig. 2c). In addition, the correlation between cell adhesion and pore size was maintained over a wide range not only below the threshold of 70 nm but also above the threshold (Fig. 2d). The pore size also affected the cell morphology (Fig. 2e), which suggests that the extracellular signals generated by the NPG substrate were transmitted inside a cell.

### Mechanism of cell adhesion reduced by NPG

**Fig. 3:**
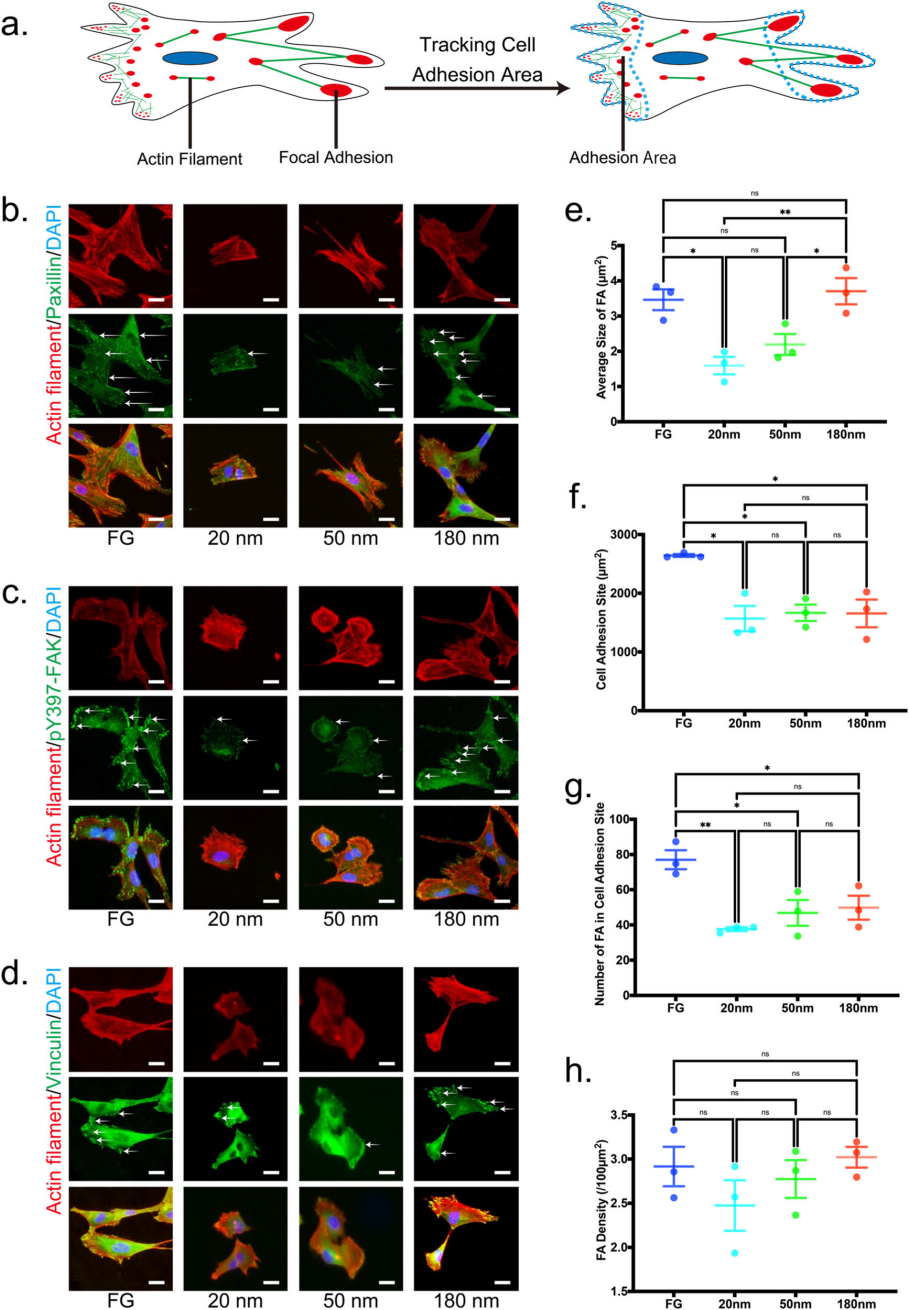
Analyses of focal adhesion (FA). **a** Schematic illustration of the analysis methods of FA. **b**–**d** Immunofluorescent staining of paxillin (green, **b**), pY397-FAK (green, **c**) and vinculin (green, **d**). Cytoskeleton and nuclei were labelled using actin filament (red, **b**–**d**) and (blue, **b**–**d**). Arrows indicate FA and integrin clustering. Scale bars for (**b**–**d**) are 10 µm. **e**–**h** Quantitative data of FA: average size of FA (**e**), cell adhesion site (**f**), number of FAs in a cell adhesion site, and (**g**) FA density (/100 µm^2^) (**h**). All data were presented as the mean ± s.e.m from *N* > 50 independent cells. The *P*-values were analyzed with one-way ANOVA followed by a Tukey’s post hoc test. * and ** indicate *P* < 0.05 and *P* < 0.01; ns indicates not significant

**Fig. 4:**
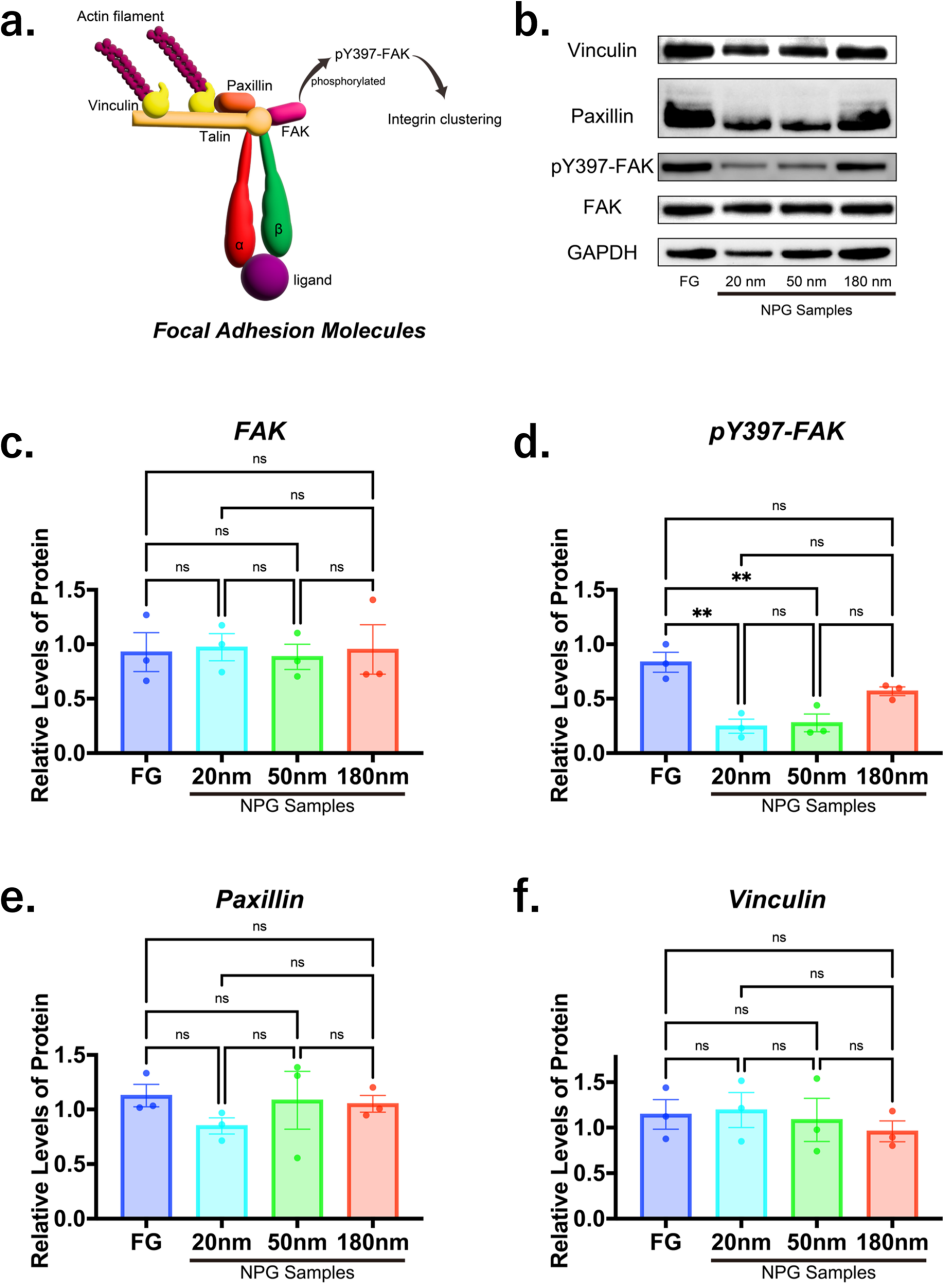
Expression levels of focal adhesion (FA) molecules. **a** Schematic illustration of FA molecules: focal adhesion kinase (FAK), phosphor Y397 (pY397)-FAK, paxillin, and vinculin were analyzed. **b**–**f** Representative western blot images (**b**) and quantitative data showing expression levels of FAK (**c**), pY397-FAK (**d**), paxillin (**e**), and vinculin (**f**). FAK was phosphorylated for FG and 180 nm NPG samples. Protein expression levels were normalized by GAPDH as an internal control. All data were presented as the mean ± s.e.m from three independent experiments. The *P*-values were analyzed with one-way ANOVA followed by a Tukey’s post hoc test. ** indicate *P* < 0.01; ns indicates not significant

FA plays a critical role in cell adhesion [11, 13, 47, 48]. Therefore, we investigated the size and density in FA. The analysis methods of FA are schematically shown in Fig. 3a. Also, paxillin, FAK and vinculin, which are main molecules in FA (Fig. 4a), were investigated by immunofluorescent staining, as shown in Fig. 3b–d. Based on the results, quantitative data of average size of FA, cell adhesion site, number of FAs in a cell adhesion site and FA density were investigated (Fig. 3e–h). Notably, the size and density in FA decreased with decreasing pore size. This corresponds to the fact that the cell adhesion was reduced with decreasing pore size.

PCR measurements showed that FA was less activated with decreasing pore size (Fig. 4c, d). Clearly, the reduced cell adhesion by NPG was related to the inactivated FA. Alternatively, the activation of paxillin and vinculin had little dependence on the pore size (Fig. 4e, f), which suggests the characteristic slip and catch bonds of integrins [28, 29] were irrelevant to the cell adhesion reduced by NPG substrates.

### Clustering of integrins

**Fig. 5:**
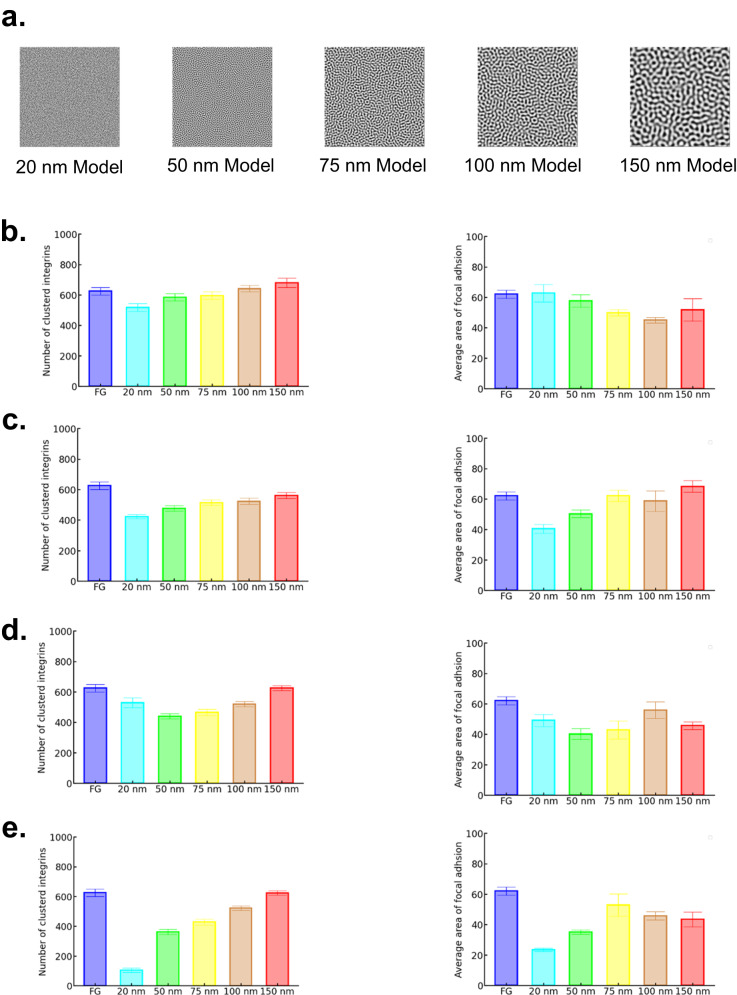
Results of Monte Carlo simulations regarding the effects of nanoporous gold (NPG) on the formation of focal adhesion by clustering of integrins. **a** Models of NPG substrates with pore sizes of 20, 50, 75, 100 and 150 nm. The substrates were modeled on the Cahn–Hilliard equation. **b**–**e** Number of clustered integrins and average area of focal adhesion in four conditions of the binding energy and the association (clustering) energy. **b** The binding energy depends on the pore size while the association energy is constant. **c** Both the binding energy and association energy depend on the pore size. **d** The two energies depend less on the pore size than the (c) condition. **e** The two energies depend more on the pore size than the (c) condition

Monte Carlo simulations with NPG models based on the Cahn-Hilliard equation (Fig. 5a) were performed to investigate why NPG affected the formation of FA by clustering of the integrins (Fig. 5). The simulations were carried out under the conditions that NPG affected only the binding energy of a single integrin to ECM and that it affected both the binding energy and the association energy of integrins because NPG definitely affects the binding energy while it does not necessarily affect the association energy (Figs. S1–S21). When NPG affected only the binding energy, the number of clustered integrins decreased with decreasing pore size; however, the FA size tended to increase with decreasing pore size (Fig. 5b). The cell adhesion depends more on the FA size than the FA density [11, 47]. Therefore, the dependence of pore size on the cell adhesion cannot be explained only from the variation in binding energy by NPG. Clustering of integrins is required for the formation of large FA [49]. Because the integrin clustering is affected by the diffusion of integrins, the high binding affinity does not necessarily lead to the coarsening of FA [46, 50].

In the case that NPG affected both the binding energy and association energy of the integrins, both the FA density and size decreased with decreasing pore size (Fig. 5c), which corresponds to the experimental facts in Fig. 3. This trend held when NPG affected the binding and association energies more (Fig. 5e), not when NPG affected the two energies less (Fig. 5d). Therefore, it is suggested that the extracellular signals generated by NPG are transmitted to specific intracellular proteins of talin and kindlin that are related to the clustering of integrins [51].

### Effects of pore size on differentiation

**Fig. 6:**
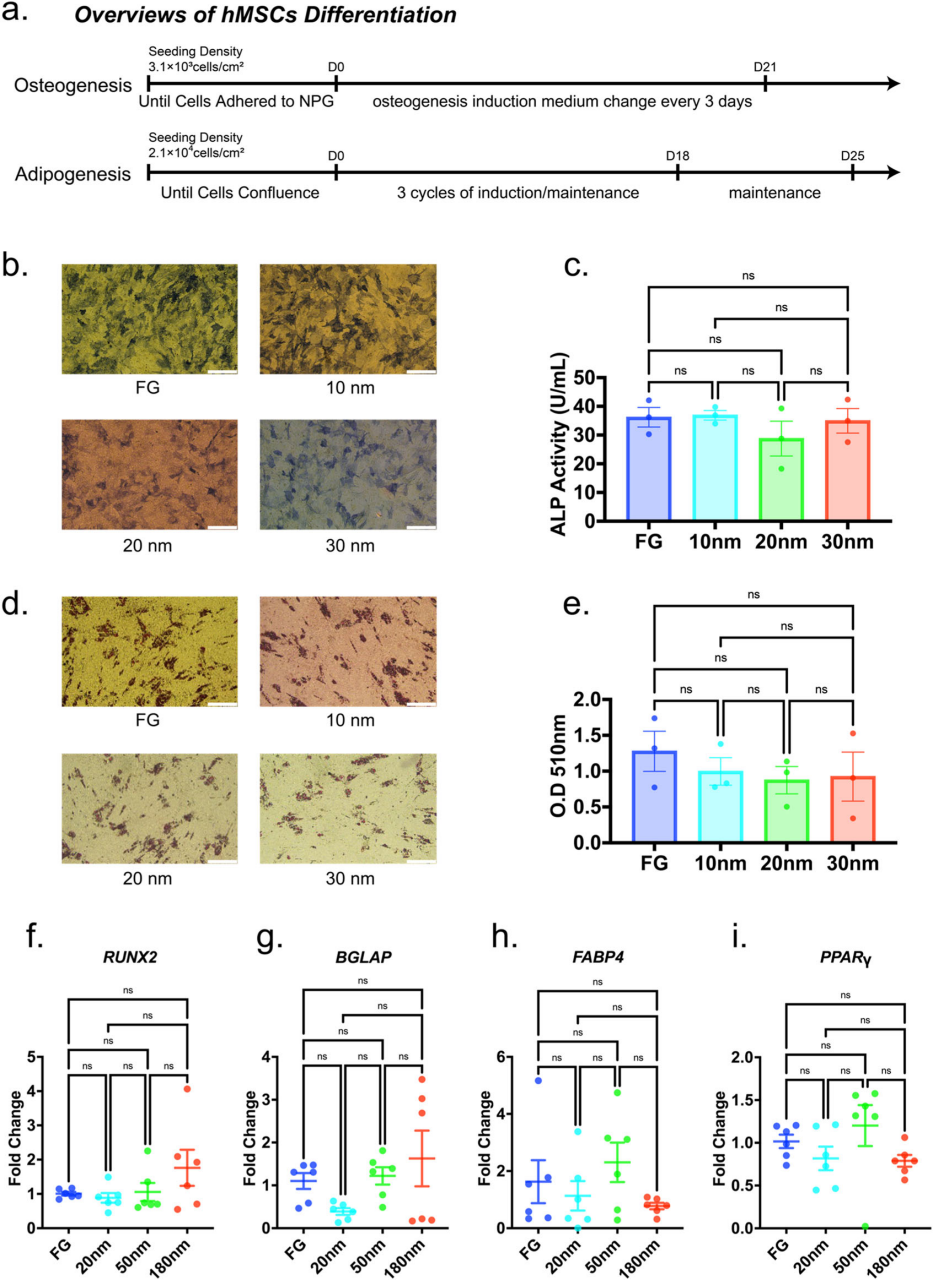
Differentiation of human mesenchymal stem cells (hMSCs). **a** Overviews and time schedules of osteogenic and adipogenic induction of hMSCs. **b**, **c** Osteogenesis of hMSCs on FG, 10, 20, and 30 nm NPG samples. Differentiations were evaluated using alkaline phosphatase staining (scale bar: 400 µm, **b**) and a quantitative assay using a microplate reader (**c**). **d**, **e** Adipogenesis of hMSCs on FG, 10, 20, and 30 nm NPG samples. Differentiations were evaluated using oil Red O staining (scale bar: 400 µm, **d**) and absorbance measurements (**e**). **f**, **g** Genetic expression of osteogenesis markers: runt-related transcription factor 2 (*RUNX2*, **f**) and bone gamma-carboxyglutamate protein (*BGLAP*, **g**). **h**, **i** Genetic expression of adipogenesis markers: fatty acid binding protein 4 (*FABP4*, **h**) and peroxisome proliferator-activated receptor γ (*PPARγ*, **i**). Genetic expressions were normalized by GAPDH as an internal control. All data were presented as the mean ± s.e.m. Data were acquired from three independent samples for (**b**–**e**), and *N* = 6 independent samples for (**f**–**i**). The *P*-values were analyzed with one-way ANOVA followed by a Tukey’s post hoc test. ns indicates not significant

Osteogenic and adipogenic differentiations of hMSCs were investigated (Fig. 6). Notably, NPG had little effect on osteogenic and adipogenic differentiation. Previous works showed that cell differentiation was affected by the surface topology [12, 13] with limited exception [52]. Thus, the present results are contrary to previous works. Zhang et al. [53] showed that the effects of pore size on osteogenic differentiation depended on the rigidity of a substrate. Generally, a substrate with a high rigidity enhances osteogenic differentiation [54]. This may be maintained when the rigidity of a substrate is on the order of less than hundreds of kPa. However, the rigidity of NPG is on the order of GPa [55]. Therefore, the difference in rigidity between FA and NPG substrates did not induce the difference in osteogenic differentiation.

## Discussion

The previous works showed that the cell adhesion was reduced with increasing topological spacing, and the threshold spacing for reduced adhesion was related to the size of talin, which plays a vital role in clustering of integrins [8, 10, 14]. Clusters of unliganded integrins bridged thin (<30 nm) matrix fibers [56], which suggests that an enhanced cell adhesion was not related to the high binding affinity of integrin to ligands, but to small topological spacing. However, the result obtained in the present work was that the cell adhesion was enhanced with increasing pore size, which is contrary to the finding shown above. In this study, the clustering of integrins and the formation of FA were activated with increasing pore size. Notably, NPG affected the intracellular proteins related to the formation of FA, such as FAK, not the proteins related to the catch bond of integrins, such as vinculin, although both FAK and vinculin can play a central role in enhancing cell adhesion by binding to talin, which directly connects to integrins. Therefore, the two signals related to cell adhesion branched off independently from FA.

**Fig. 7:**
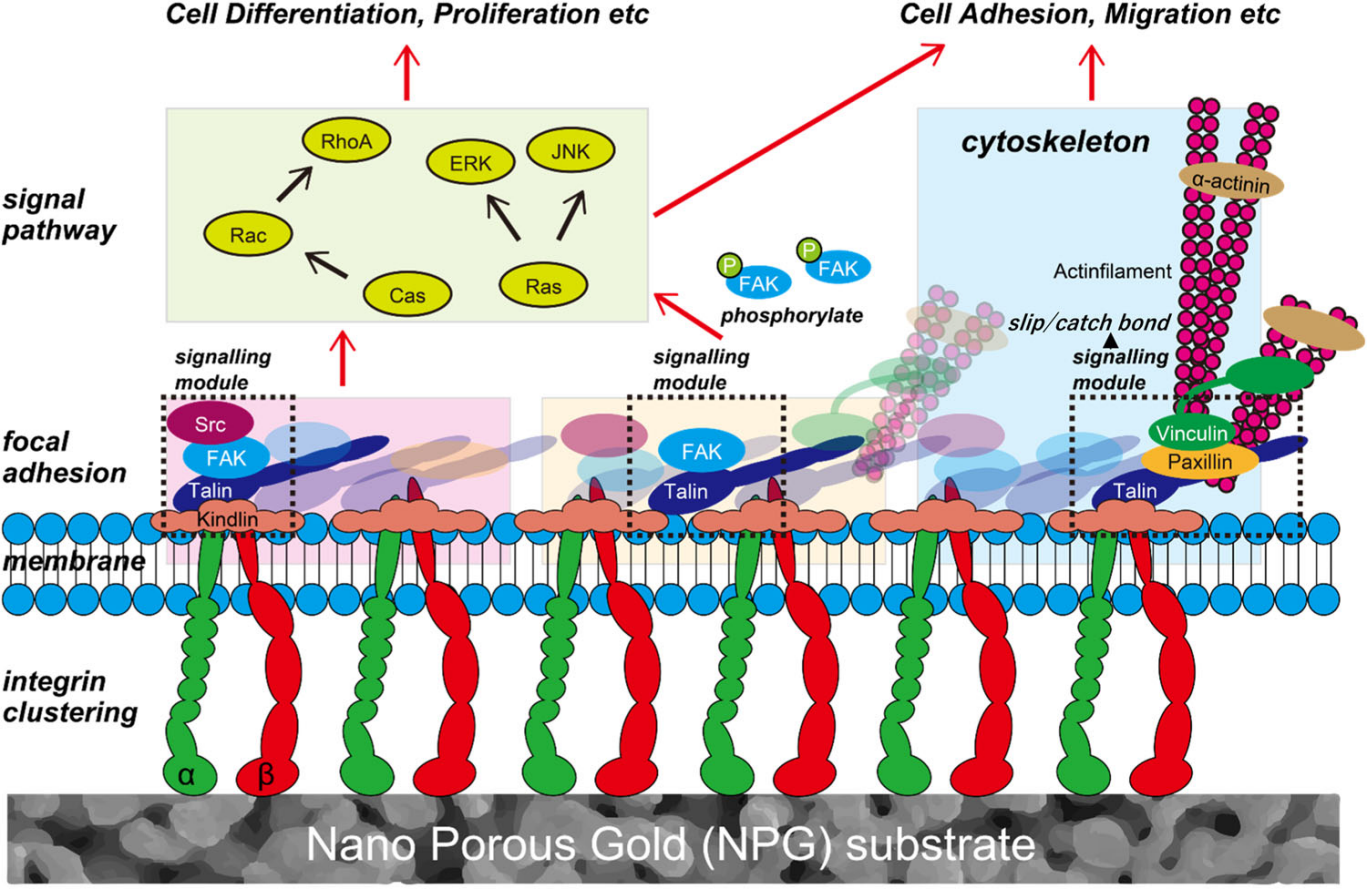
Schematic illustration of multi-branch signaling of focal adhesions (FAs) connecting to integrins. FA comprises signaling modules. Because various extracellular signals related to cell adhesion, differentiation and so on are transmitted through different modules of FA, they are independently transmitted from the integrin to inside a cell

Stutchbury et al. [57] showed that FA comprises three modules with respect to mechanotransduction: the mechanosignaling module, mechanosensing structural module, and intermediated module. Thus, FA is an aggregate of modules comprising specific proteins such as FAK, talin, and vinculin. Signals through the integrin branch off from FA to various pathways [58–64]. Therefore, it is suggested that modules play an important role in branching off. Extracellular signaling for differentiation is intracellularly transmitted through FA connecting to integrin, and it enters the cell nucleus through pathways such as ERK, JUN, and PhoA [63–71]. The present work demonstrated that NPG affected the cell adhesion through FA, whereas it had little effect on the differentiation whose signals are transmitted through FA. Therefore, the signals for cell adhesion and differentiation can be independent of each other because of transmission through different modules of FA. Collectively, FA branches off various signals through its modules, and therefore various extracellular signals related to cell adhesion, differentiation and so on can be independently transmitted from integrins to inside a cell (Fig. 7). However, note that extracellular signals related to cell adhesion and differentiation are often dependently transmitted from integrins. It is therefore suggested that extracellular signals can be independently transmitted through FA modules, depending on the circumstances.

The present work showed that the effects of NPG depended on the pore size. However, Champman et al. [33] showed that cell adhesion was independent of the pore size. This contradicting result may be attributed to the difference in cell type because the activated integrin depends on the cell type [72], or to the lesser effect of NPG on the binding and clustering energies, as shown in Fig. 5d. In the present work, different cell types of HFs for the cell adhesion measurements and hMSCs for the differentiation measurements were used. However, HFs and hMSCs are non-epithelial and hMSCs can be differentiated to be HFs. Hence, the tendency of cell adhesion of hMSCs is probably similar to that of HFs.

## Conclusions

NPG substrates with pore sizes of approximately 10, 20, 30, 50, and 180 nm were fabricated and their effects on cell adhesion and osteogenic and adipogenic differentiation were investigated. The cell adhesion was reduced with decreasing pore size, which is contrary to previous results where the cell adhesion was enhanced with decreasing topological spacing. The decrease in cell adhesion with decreasing pore size was explained by less activated FAs.

NPG substrates had little effect on differentiation, which suggests that signals for cell adhesion and differentiation can be independently transmitted through FA modules.

### Supplementary information

Supple.data

## Data Availability

The data that support the findings of this study are available from the corresponding author upon reasonable request.

## Code availability

The data that support the findings of this study are available from the corresponding author upon reasonable request.
