# Supplementary material for: Unusual effects of a nanoporous gold substrate on cell adhesion and differentiation because of independent multi-branch signaling of focal adhesions
Source: J Mater Sci Mater Med. 2023 Oct 26;34(11):54. doi: 10.1007/s10856-023-06760-0 (PMC10602965; doi:10.1007/s10856-023-06760-0)
Supplement: Supplementary file 1 — Supple.data [file 10856_2023_6760_MOESM1_ESM.docx]

**Supplementary Information**

**S1. Heat treatment for nanoporous Au**

Table S1. Heat treatment conditions for nanoporous Au.

| Process  Samples | **Dealloying Process** | | **Heat Treatment** | |
| --- | --- | --- | --- | --- |
|  | Temperature (℃) | Time (h) | Temperature (℃) | Time (h) |
| 10 nm Samples | -20 | 24 |  |  |
| 20 nm Samples | 25 | 24 |  |  |
| 30 nm Samples | 25 | 48 |  |  |
| 50 nm Samples | -20 | 24 | 200 | 3 |
| 180 nm Samples | -20 | 24 | 250 | 6 |

**S2. Monte Carlo simulations of clustering of integrins**

**
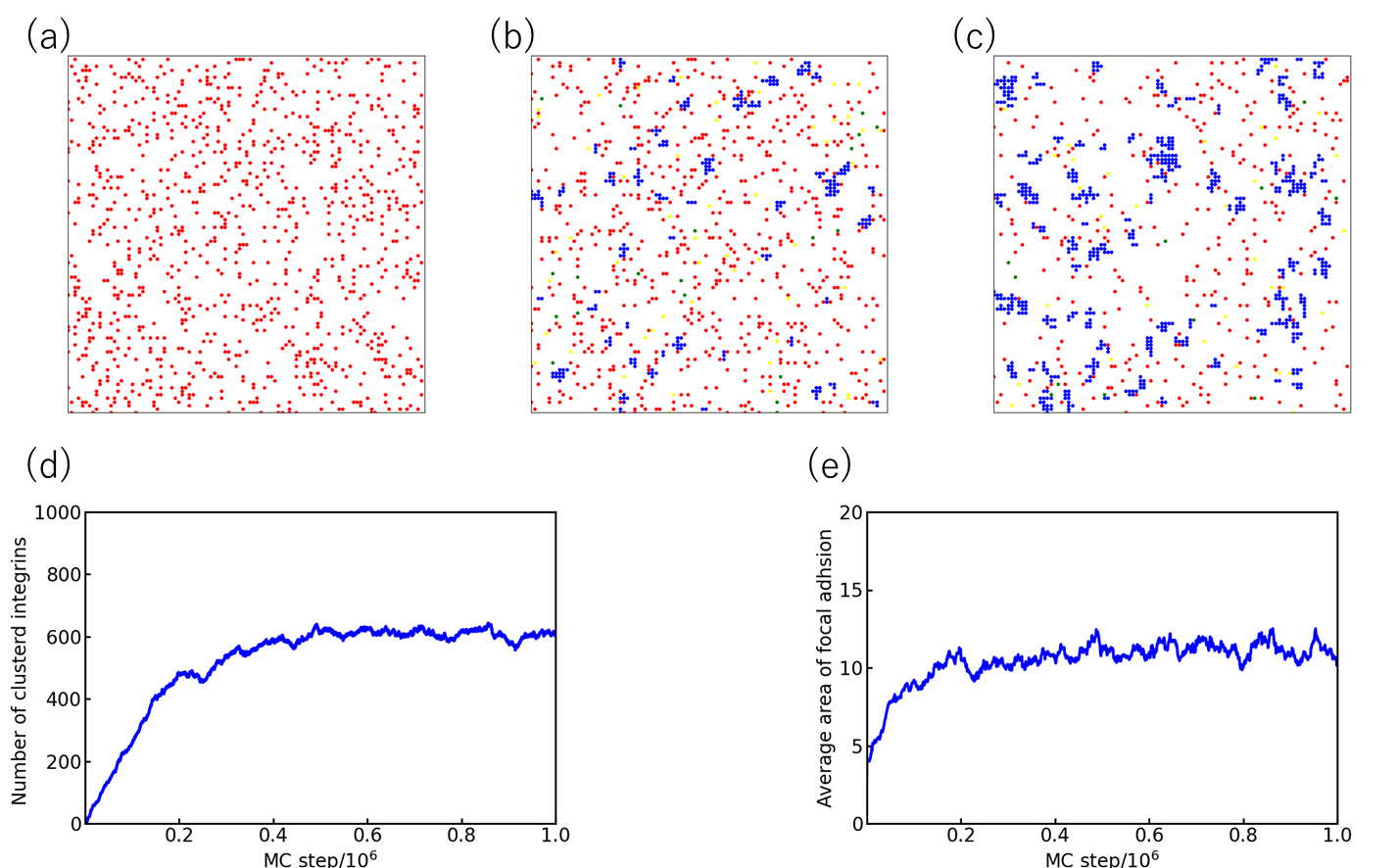
**

**Fig. S1. Variations in integrin clustering with simulation time for nanoporous Au with the pore size of 20 nm where the binding energy depend on the pore size, *α* = 1 and** $\boldsymbol{E}_{\boldsymbol{c}}\boldsymbol{=6.2}$**.** (a) MS step = 0. (b) MS step = 100000. (c) MS step = 800000. Red points show non-clustered integrins and blue points show clustered integrins. (d) Variation in number of clustered integrins. (e) Variation in average area of focal adhesion.

**
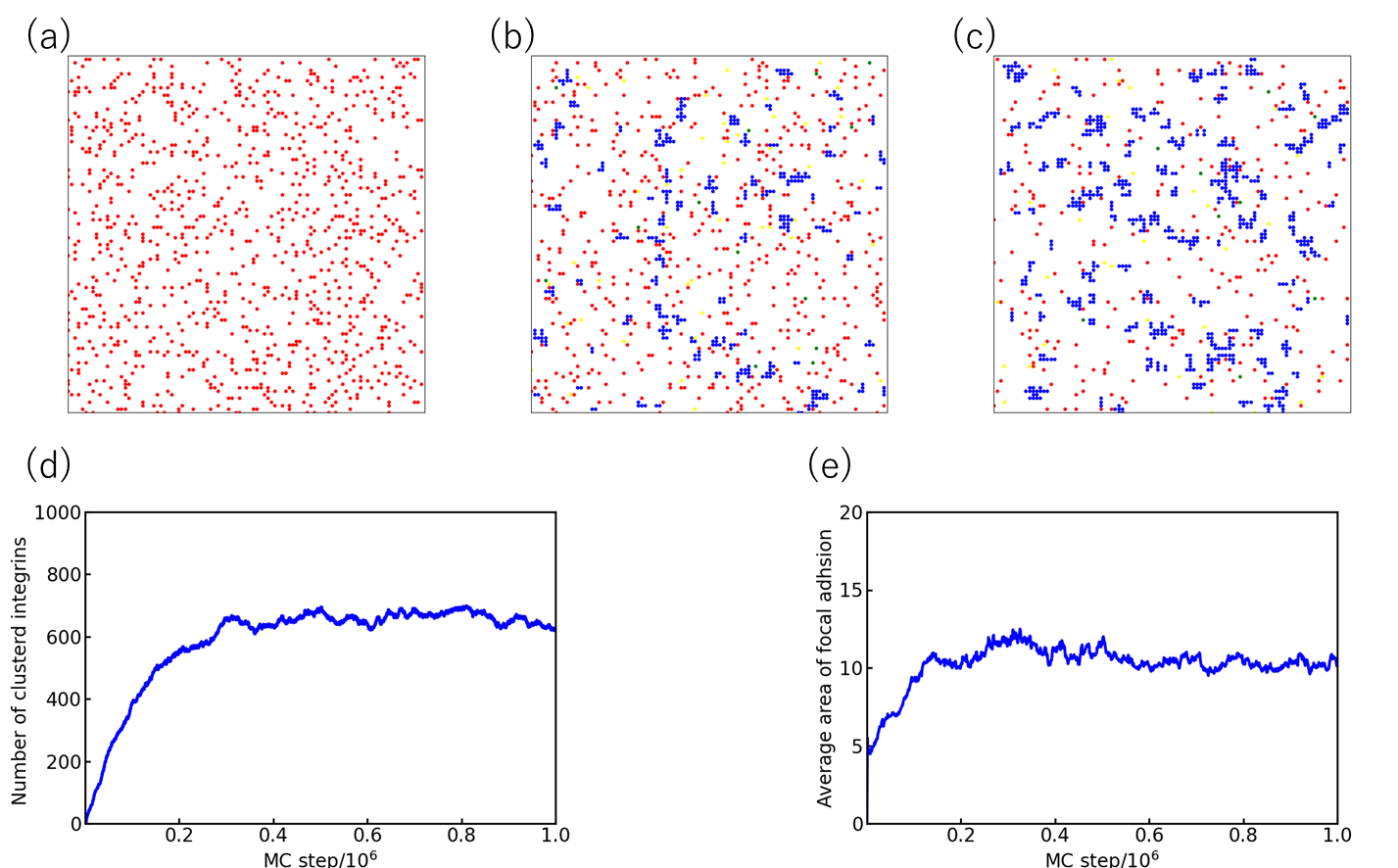
**

**Fig. S2. Variations in integrin clustering with simulation time for nanoporous Au with the pore size of 50 nm where the binding energy depend on the pore size, *α* = 1 and** $\boldsymbol{E}_{\boldsymbol{c}}\boldsymbol{=6.2}$**.** (a) MS step = 0. (b) MS step = 100000. (c) MS step = 800000. Red points show non-clustered integrins and blue points show clustered integrins. (d) Variation in number of clustered integrins. (e) Variation in average area of focal adhesion.

**
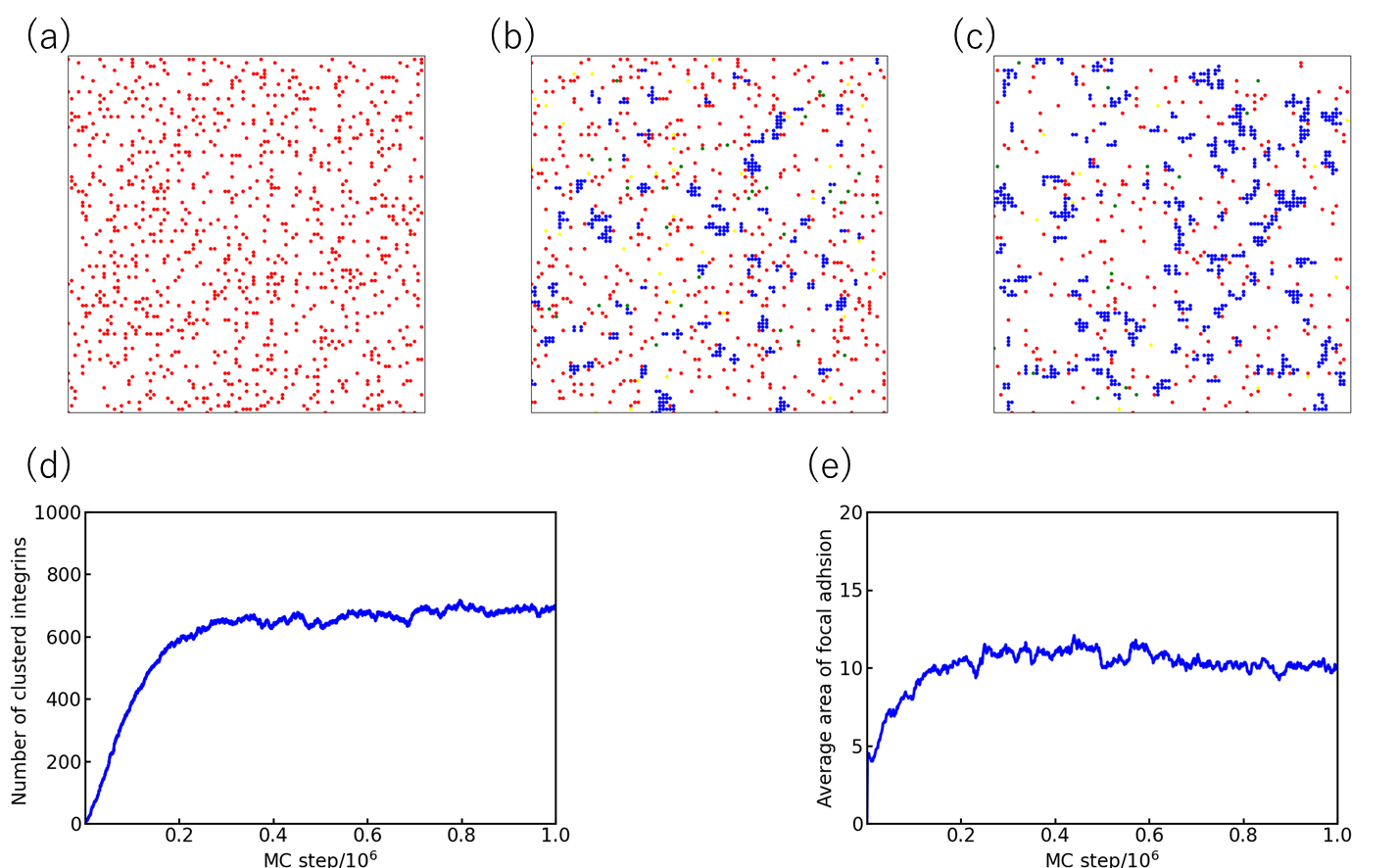
**

**Fig. S3. Variations in integrin clustering with simulation time for nanoporous Au with the pore size of 75 nm where the binding energy depend on the pore size, *α* = 1 and** $\boldsymbol{E}_{\boldsymbol{c}}\boldsymbol{=6.2}$**.** (a) MS step = 0. (b) MS step = 100000. (c) MS step = 800000. Red points show non-clustered integrins and blue points show clustered integrins. (d) Variation in number of clustered integrins. (e) Variation in average area of focal adhesion.

**
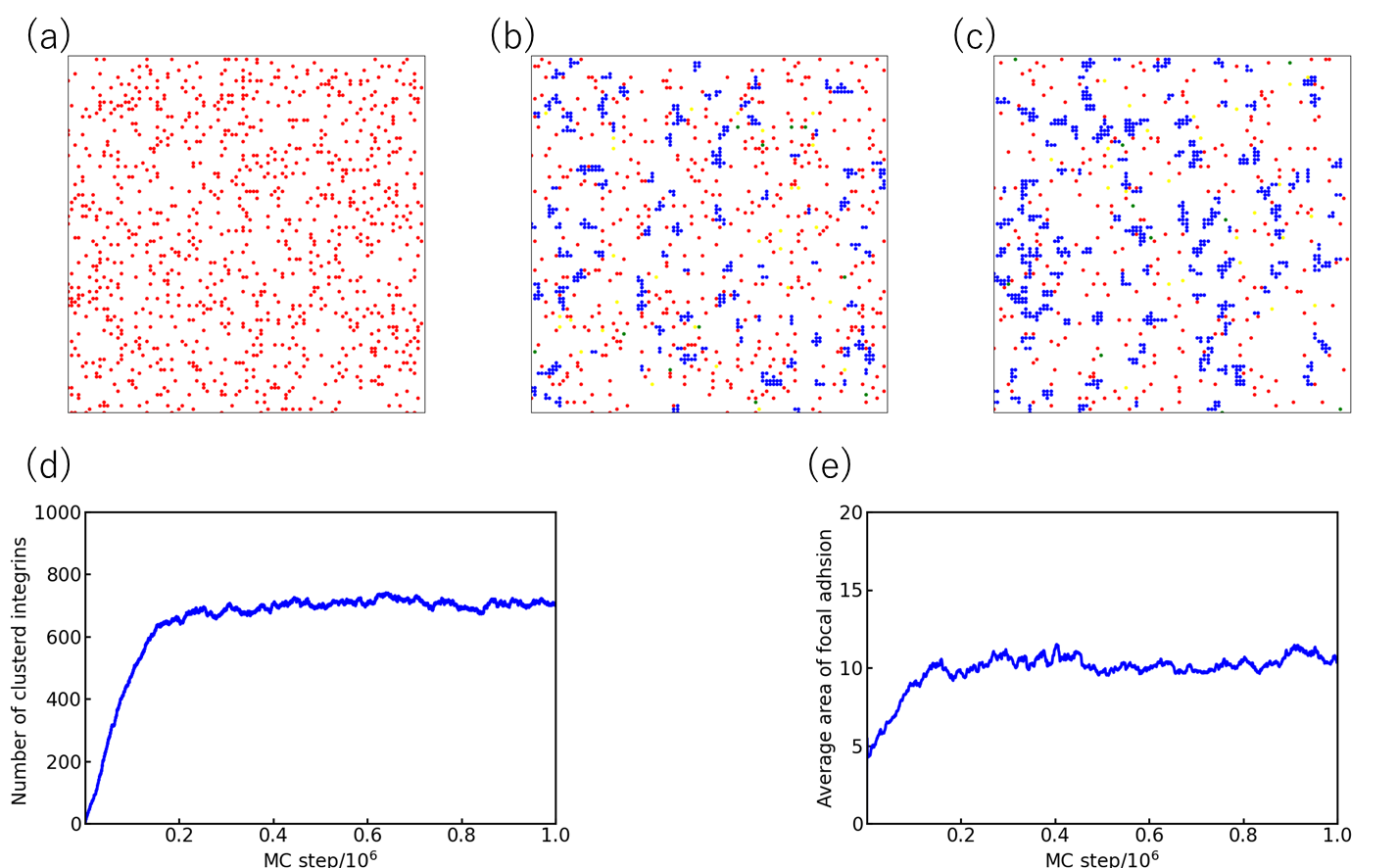
**

**Fig. S4. Variations in integrin clustering with simulation time for nanoporous Au with the pore size of 100 nm where the binding energy depend on the pore size, *α* = 1 and** $\boldsymbol{E}_{\boldsymbol{c}}\boldsymbol{=6.2}$**.** (a) MS step = 0. (b) MS step = 100000. (c) MS step = 800000. Red points show non-clustered integrins and blue points show clustered integrins. (d) Variation in number of clustered integrins. (e) Variation in average area of focal adhesion.

**
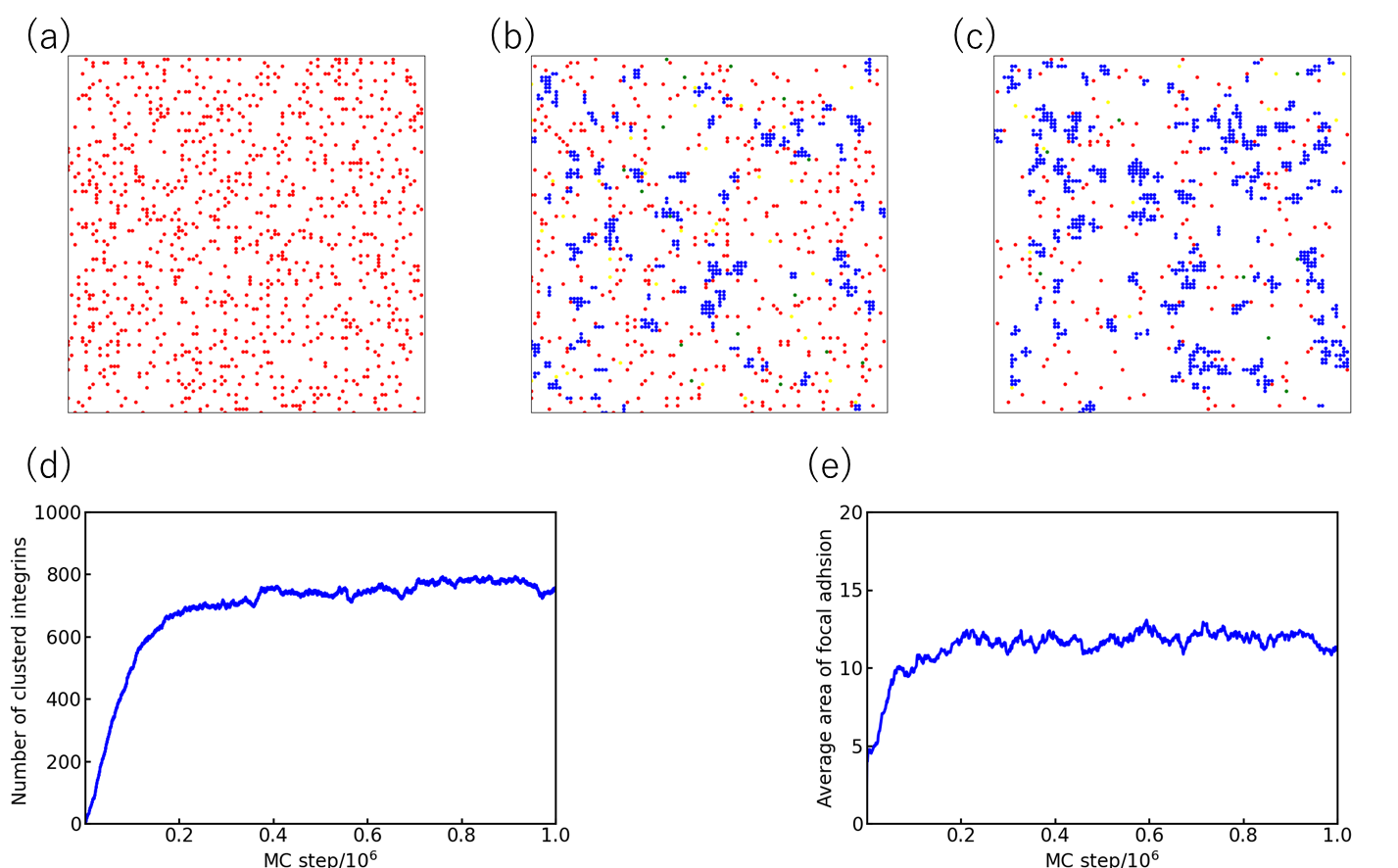
**

**Fig. S5. Variations in integrin clustering with simulation time for nanoporous Au with the pore size of 150 nm where the binding energy depend on the pore size, *α* = 1 and** $\boldsymbol{E}_{\boldsymbol{c}}\boldsymbol{=6.2}$**.** (a) MS step = 0. (b) MS step = 100000. (c) MS step = 800000. Red points show non-clustered integrins and blue points show clustered integrins. (d) Variation in number of clustered integrins. (e) Variation in average area of focal adhesion.

**
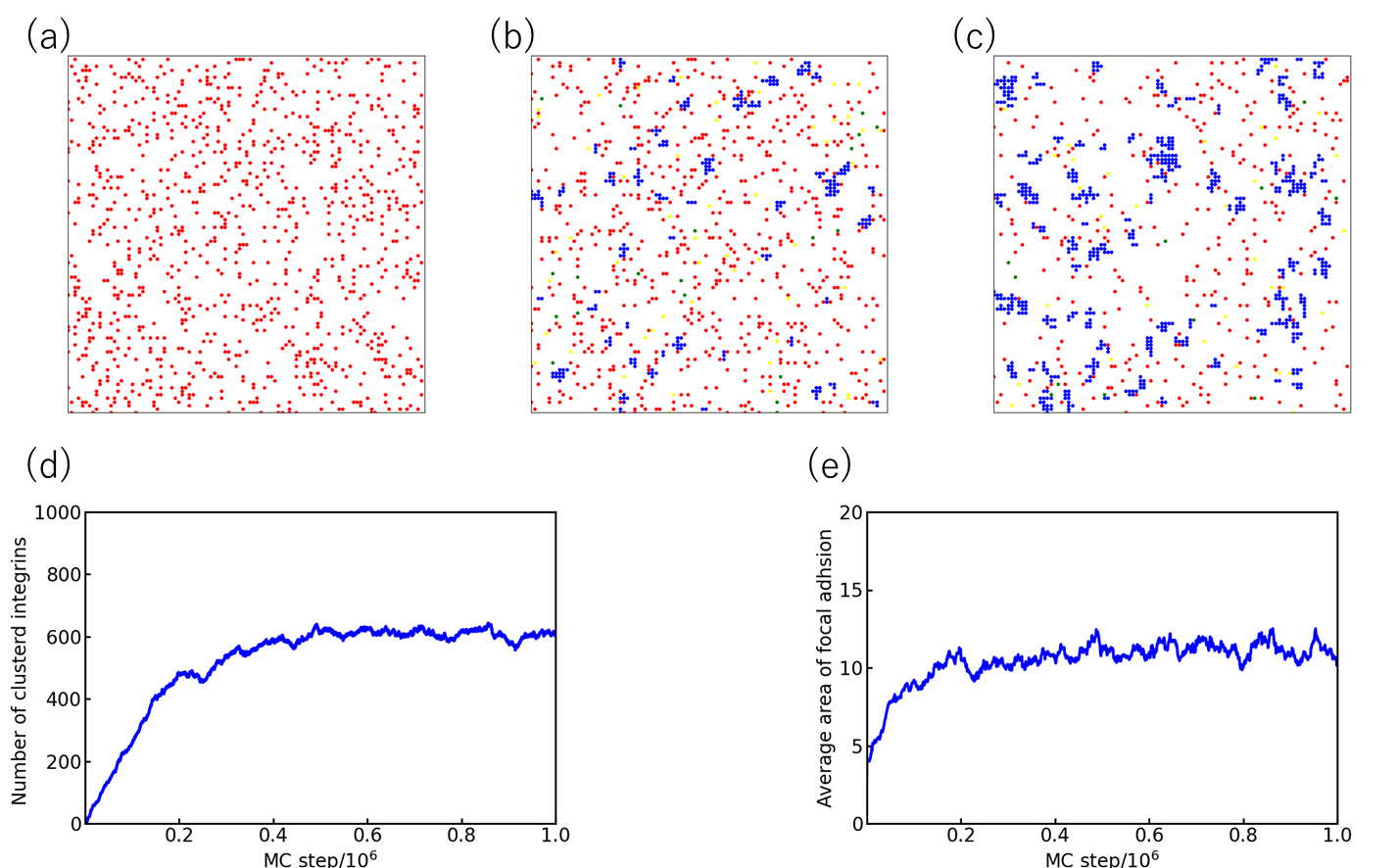
**

**Fig. S6. Variations in integrin clustering with simulation time for nanoporous Au with the pore size of 20 nm where the binding energy and the association energy depend on the pore size, *α* = 1 and *β* = 1.** (a) MS step = 0. (b) MS step = 100000. (c) MS step = 800000. Red points show non-clustered integrins and blue points show clustered integrins. (d) Variation in number of clustered integrins. (e) Variation in average area of focal adhesion.

**
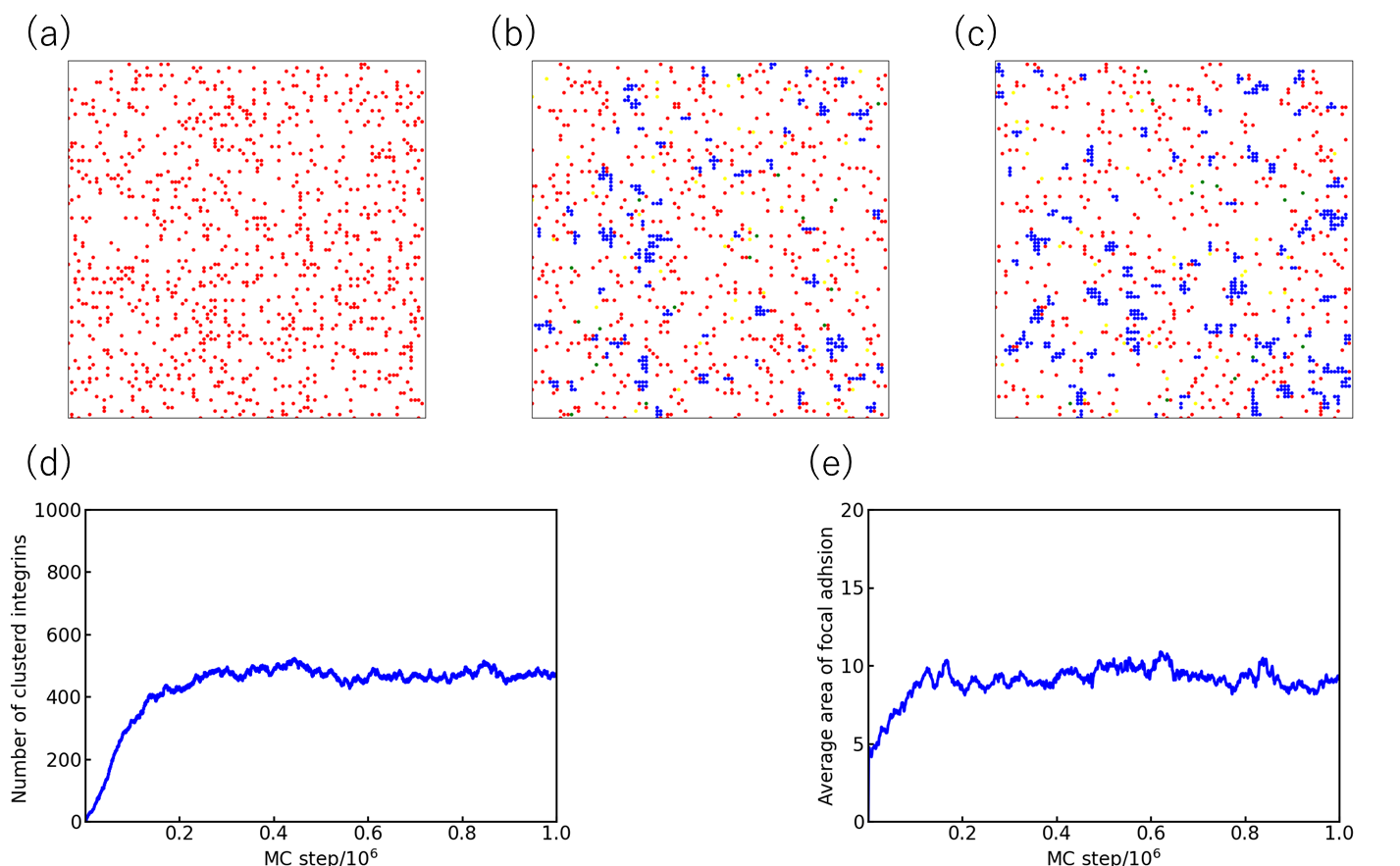
**

**Fig. S7. Variations in integrin clustering with simulation time for nanoporous Au with the pore size of 50 nm where the binding energy and the association energy depend on the pore size, *α* = 1 and *β* = 1.** (a) MS step = 0. (b) MS step = 100000. (c) MS step = 800000. Red points show non-clustered integrins and blue points show clustered integrins. (d) Variation in number of clustered integrins. (e) Variation in average area of focal adhesion.

**
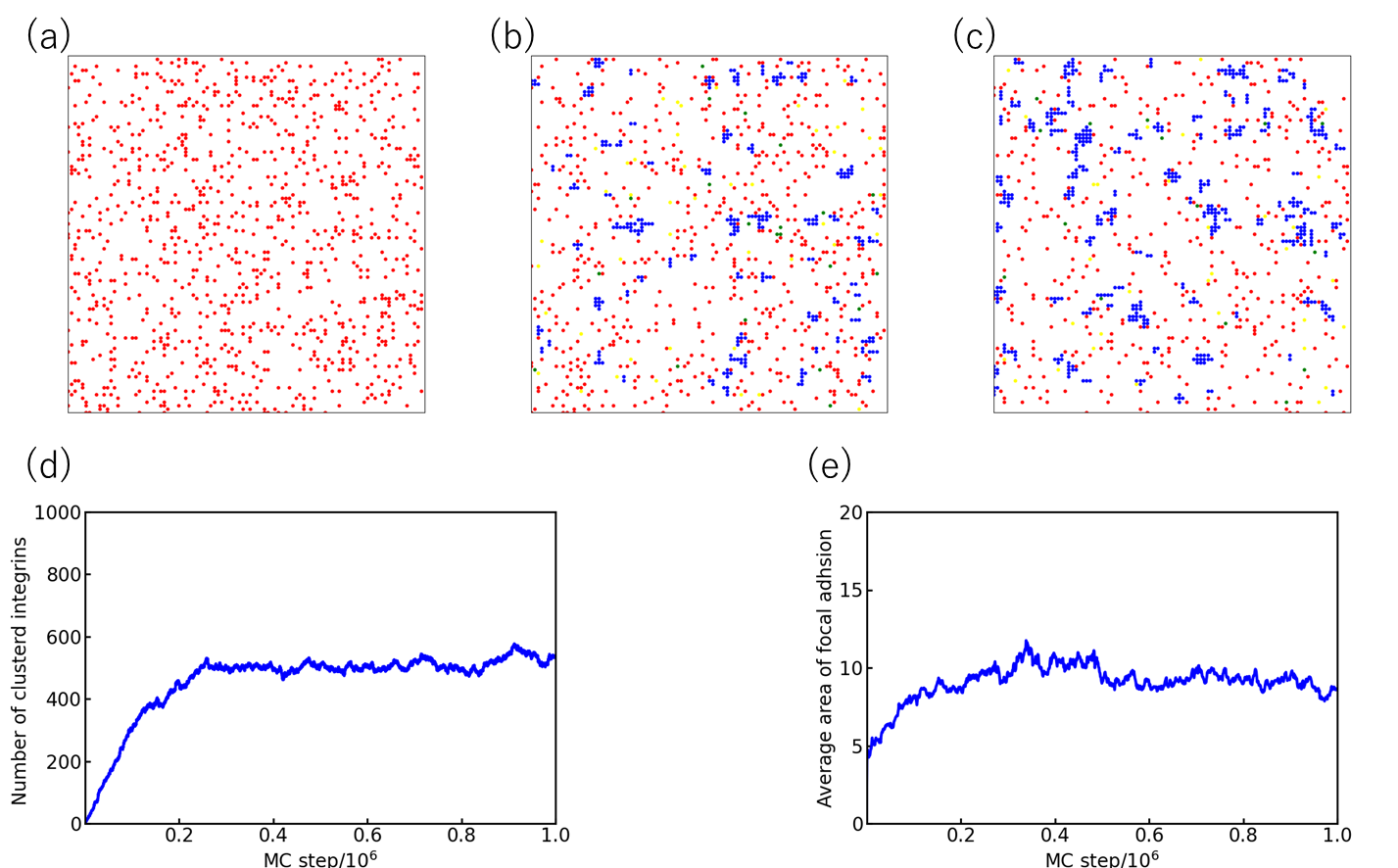
**

**Fig. S8. Variations in integrin clustering with simulation time for nanoporous Au with the pore size of 75 nm where the binding energy and the association energy depend on the pore size, *α* = 1 and *β* = 1.** (a) MS step = 0. (b) MS step = 100000. (c) MS step = 800000. Red points show non-clustered integrins and blue points show clustered integrins. (d) Variation in number of clustered integrins. (e) Variation in average area of focal adhesion.

**
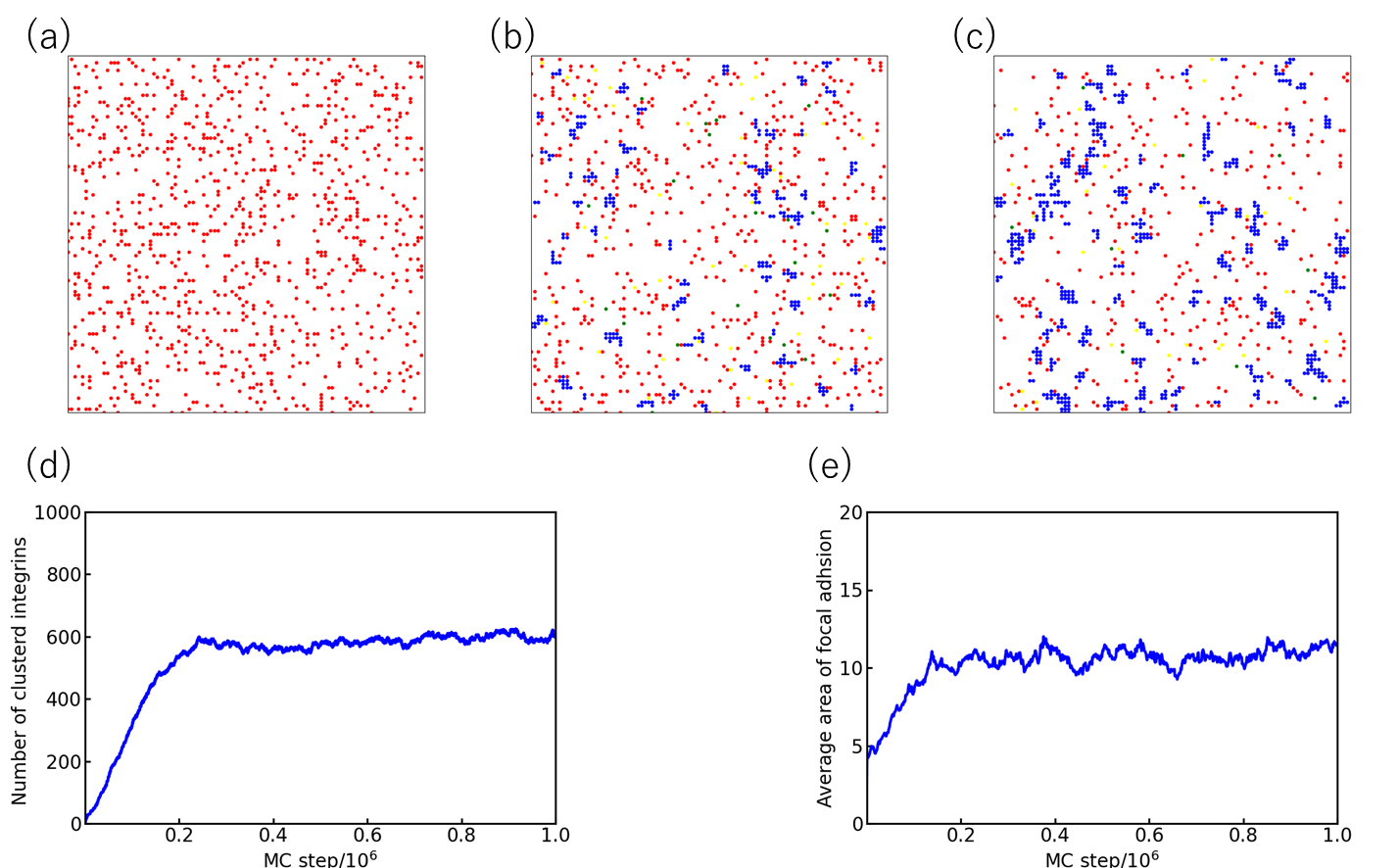
**

**Fig. S9. Variations in integrin clustering with simulation time for nanoporous Au with the pore size of 100 nm where the binding energy and the association energy depend on the pore size, *α* = 1 and *β* = 1.** (a) MS step = 0. (b) MS step = 100000. (c) MS step = 800000. Red points show non-clustered integrins and blue points show clustered integrins. (d) Variation in number of clustered integrins. (e) Variation in average area of focal adhesion.

**
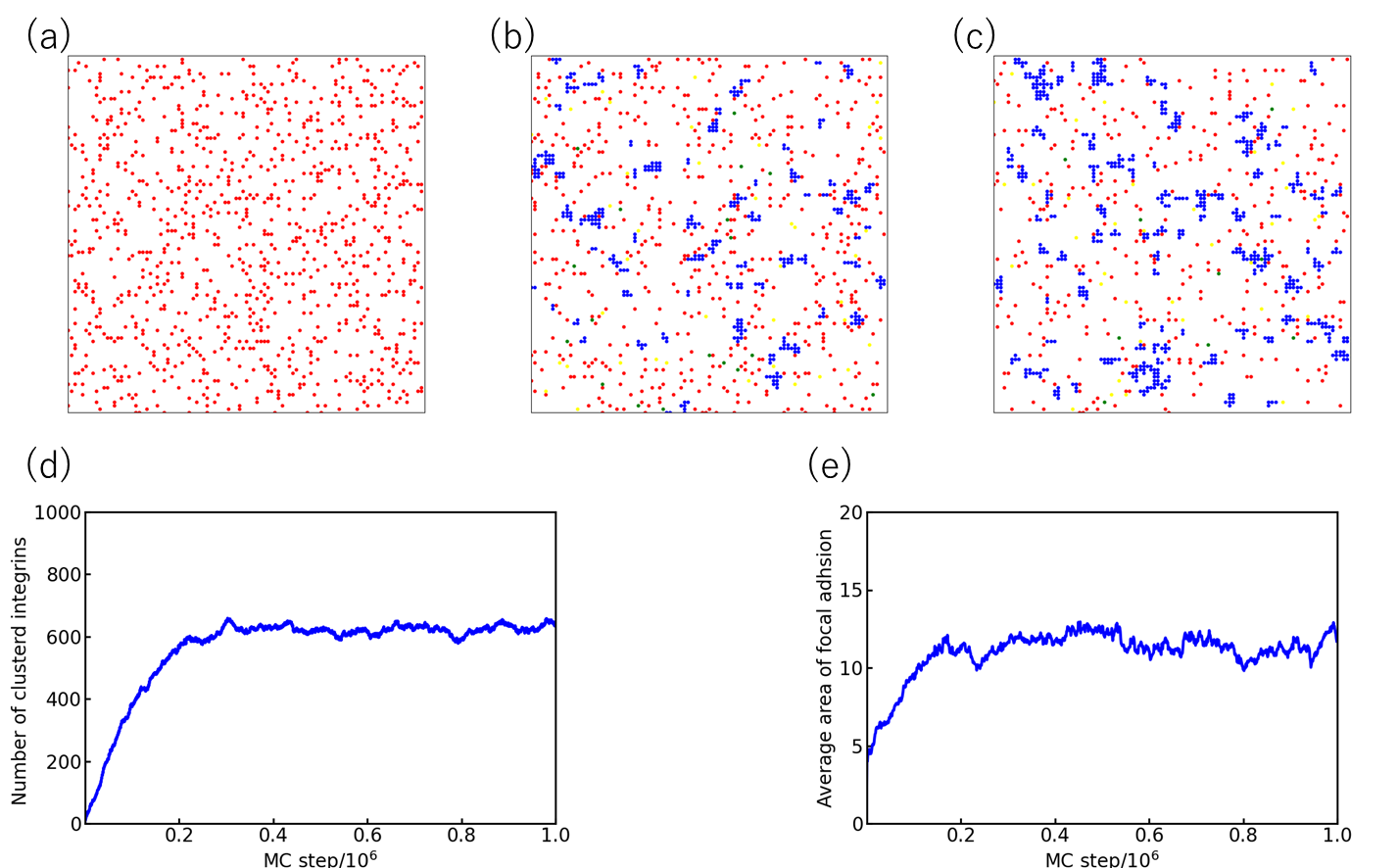
**

**Fig. S10. Variations in integrin clustering with simulation time for nanoporous Au with the pore size of 150 nm where the binding energy and the association energy depend on the pore size, *α* = 1 and *β* = 1.** (a) MS step = 0. (b) MS step = 100000. (c) MS step = 800000. Red points show non-clustered integrins and blue points show clustered integrins. (d) Variation in number of clustered integrins. (e) Variation in average area of focal adhesion.

**
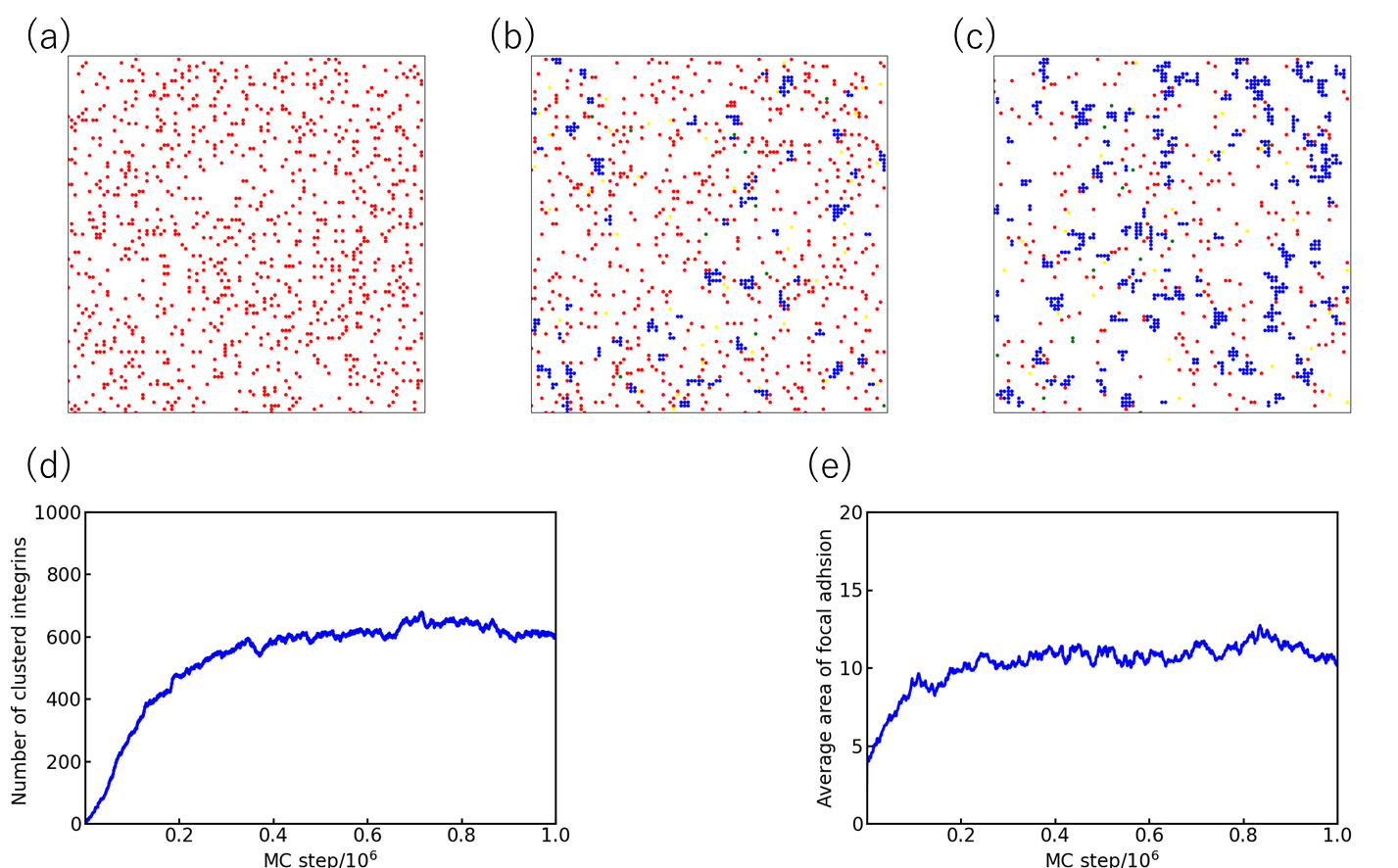
**

**Fig. S11. Variations in integrin clustering with simulation time for nanoporous Au with the pore size of 20 nm where the binding energy and the association energy depend on the pore size, *α* = 0.5 and *β* = 0.5.** (a) MS step = 0. (b) MS step = 100000. (c) MS step = 800000. Red points show non-clustered integrins and blue points show clustered integrins. (d) Variation in number of clustered integrins. (e) Variation in average area of focal adhesion.

**
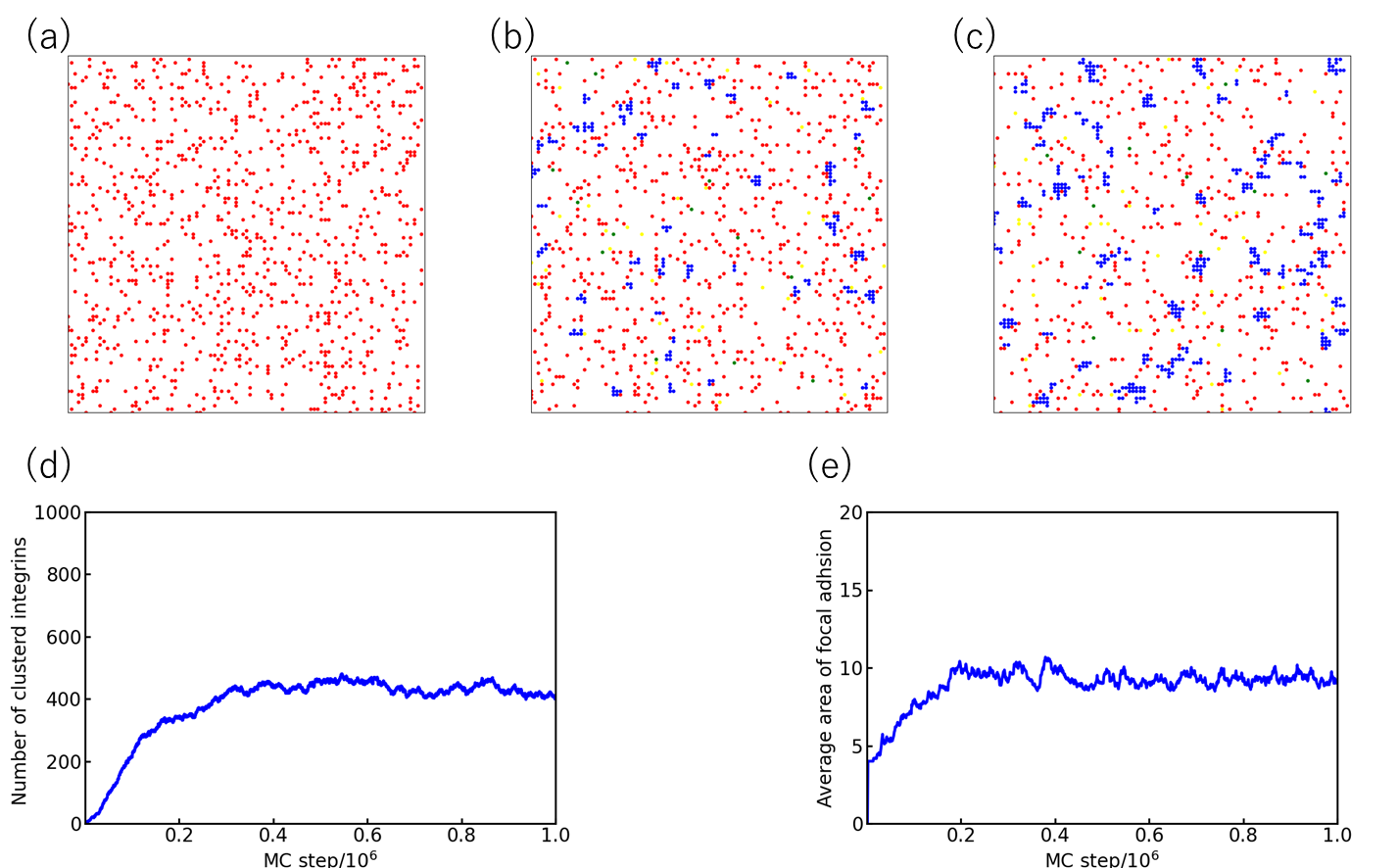
**

**Fig. S12. Variations in integrin clustering with simulation time for nanoporous Au with the pore size of 50 nm where the binding energy and the association energy depend on the pore size, *α* = 0.5 and *β* = 0.5.** (a) MS step = 0. (b) MS step = 100000. (c) MS step = 800000. Red points show non-clustered integrins and blue points show clustered integrins. (d) Variation in number of clustered integrins. (e) Variation in average area of focal adhesion.

**
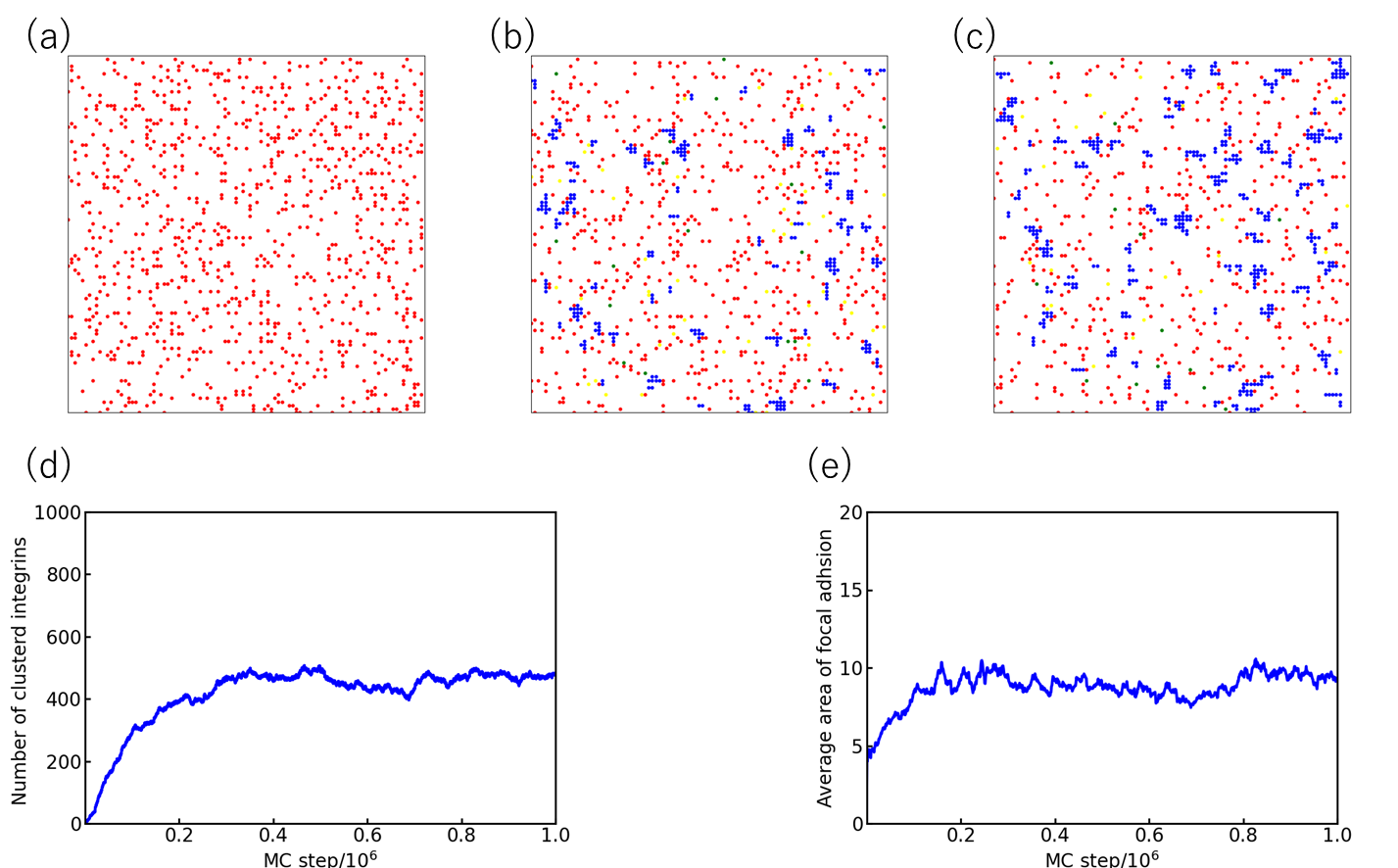
**

**Fig. S13. Variations in integrin clustering with simulation time for nanoporous Au with the pore size of 75 nm where the binding energy and the association energy depend on the pore size, *α* = 0.5 and *β* = 0.5.** (a) MS step = 0. (b) MS step = 100000. (c) MS step = 800000. Red points show non-clustered integrins and blue points show clustered integrins. (d) Variation in number of clustered integrins. (e) Variation in average area of focal adhesion

**
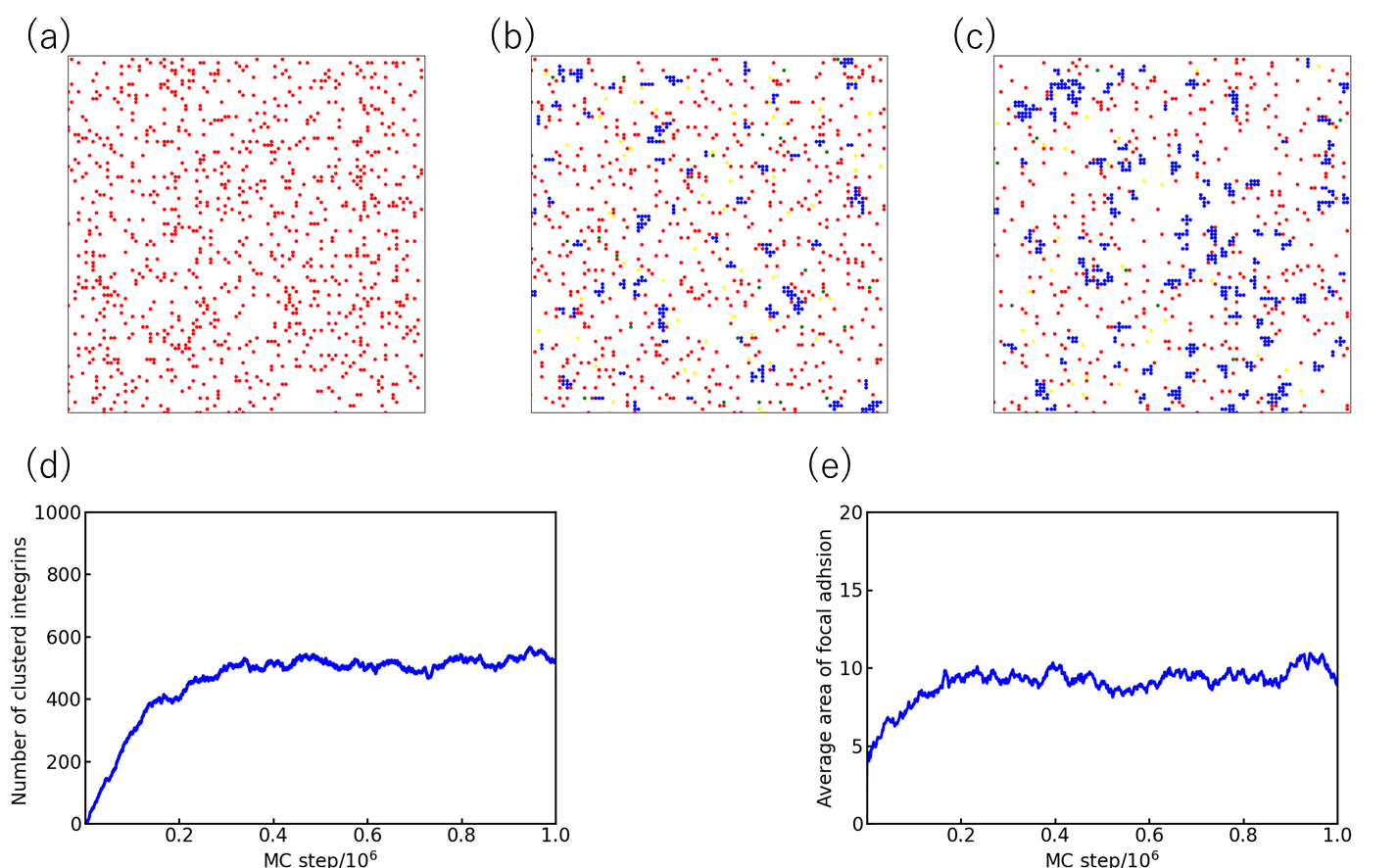
**

**Fig. S14. Variations in integrin clustering with simulation time for nanoporous Au with the pore size of 100 nm where the binding energy and the association energy depend on the pore size, *α* = 0.5 and *β* = 0.5.** (a) MS step = 0. (b) MS step = 100000. (c) MS step = 800000. Red points show non-clustered integrins and blue points show clustered integrins. (d) Variation in number of clustered integrins. (e) Variation in average area of focal adhesion

**
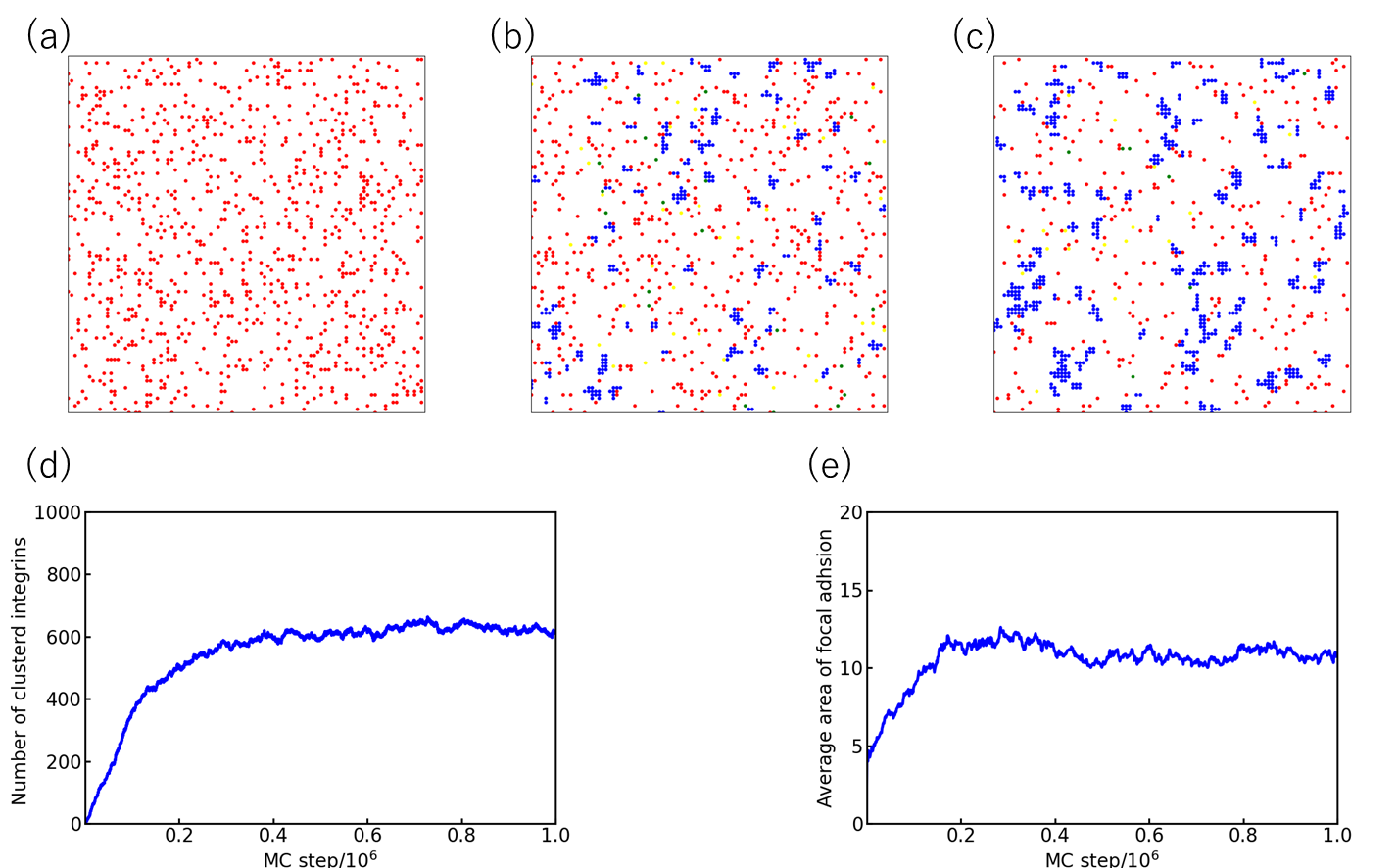
Fig. S15. Variations in integrin clustering with simulation time for nanoporous Au with the pore size of 150 nm where the binding energy and the association energy depend on the pore size, *α* = 0.5 and *β* = 0.5.** (a) MS step = 0. (b) MS step = 100000. (c) MS step = 800000. Red points show non-clustered integrins and blue points show clustered integrins. (d) Variation in number of clustered integrins. (e) Variation in average area of focal adhesion

**
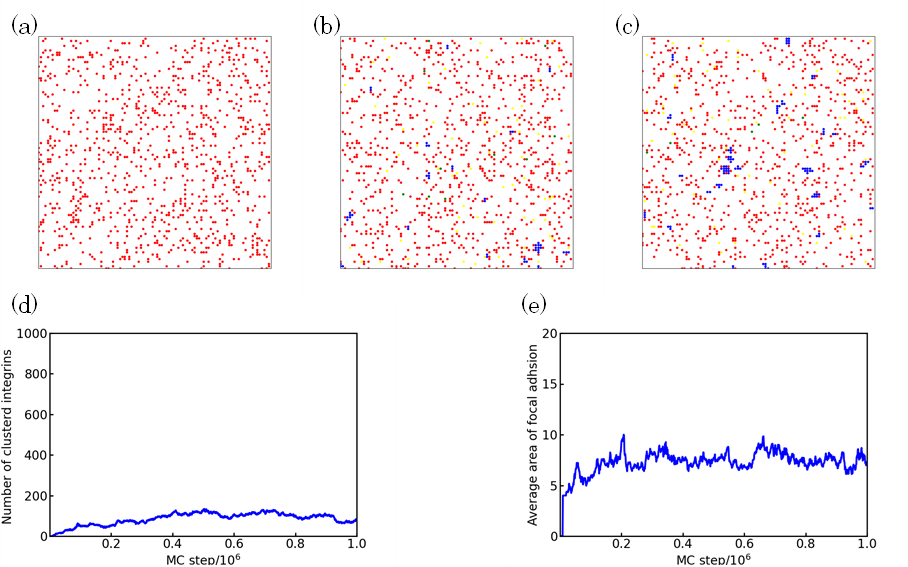
**

**Fig. S16. Variations in integrin clustering with simulation time for nanoporous Au with the pore size of 20 nm where the binding energy and the association energy depend on the pore size, *α* = 2.5 and *β* = 2.0.** (a) MS step = 0. (b) MS step = 100000. (c) MS step = 800000. Red points show non-clustered integrins and blue points show clustered integrins. (d) Variation in number of clustered integrins. (e) Variation in average area of focal adhesion

**
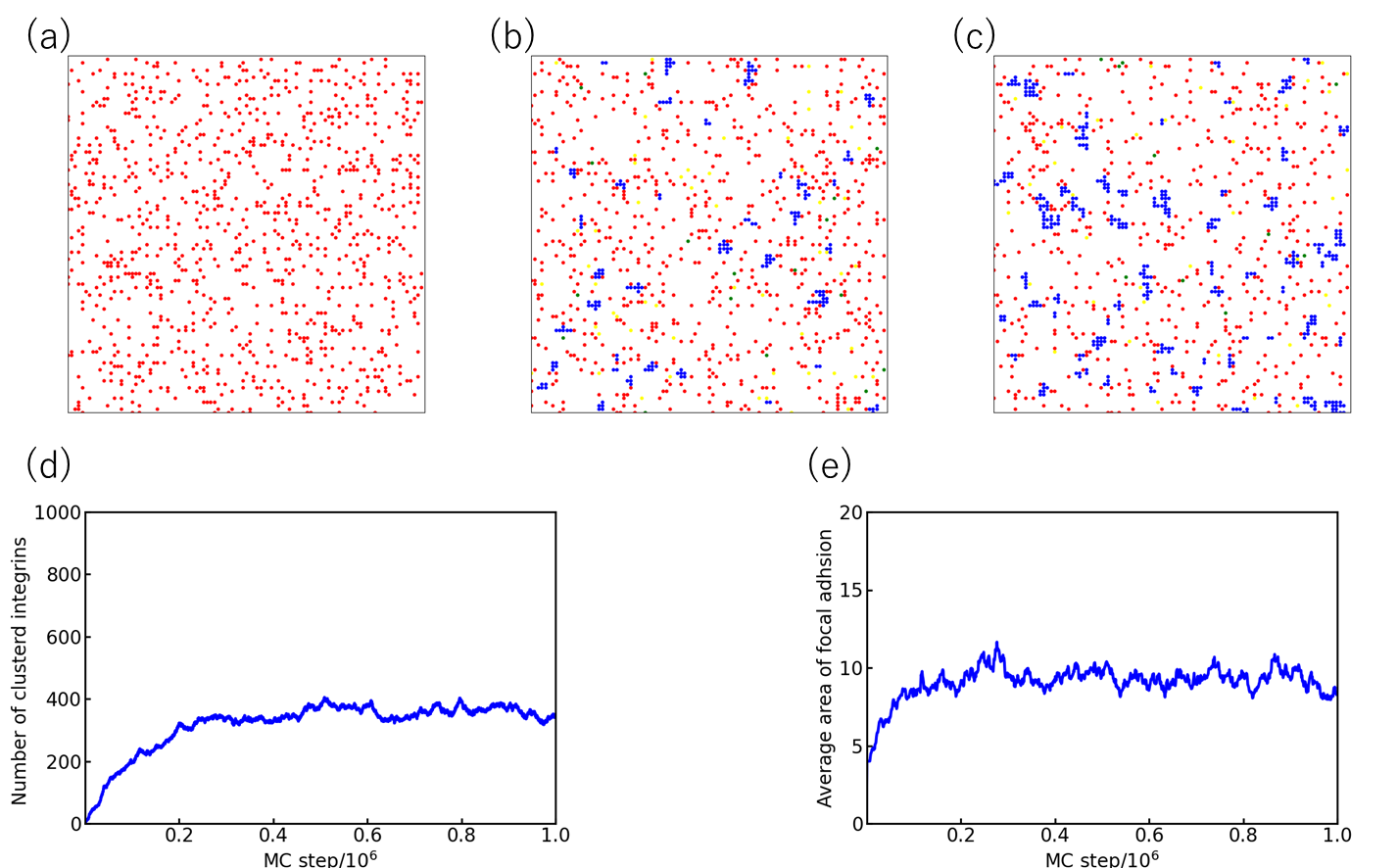
**

**Fig. S17. Variations in integrin clustering with simulation time for nanoporous Au with the pore size of 50 nm where the binding energy and the association energy depend on the pore size, *α* = 2.5 and *β* = 2.0.** (a) MS step = 0. (b) MS step = 100000. (c) MS step = 800000. Red points show non-clustered integrins and blue points show clustered integrins. (d) Variation in number of clustered integrins. (e) Variation in average area of focal adhesion

**
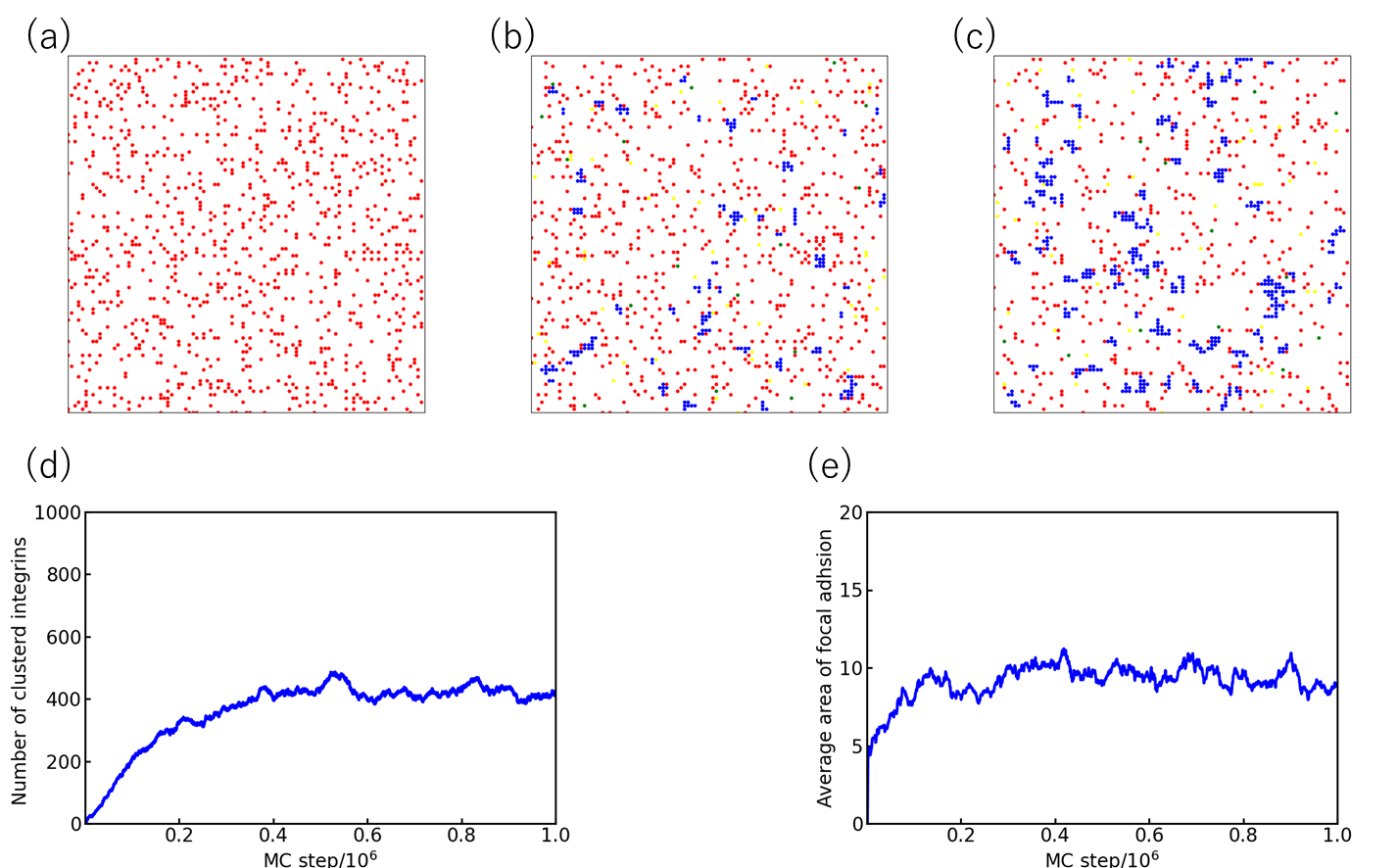
**

**Fig. S18. Variations in integrin clustering with simulation time for nanoporous Au with the pore size of 75 nm where the binding energy and the association energy depend on the pore size, *α* = 2.5 and *β* = 2.0.** (a) MS step = 0. (b) MS step = 100000. (c) MS step = 800000. Red points show non-clustered integrins and blue points show clustered integrins. (d) Variation in number of clustered integrins. (e) Variation in average area of focal adhesion

**
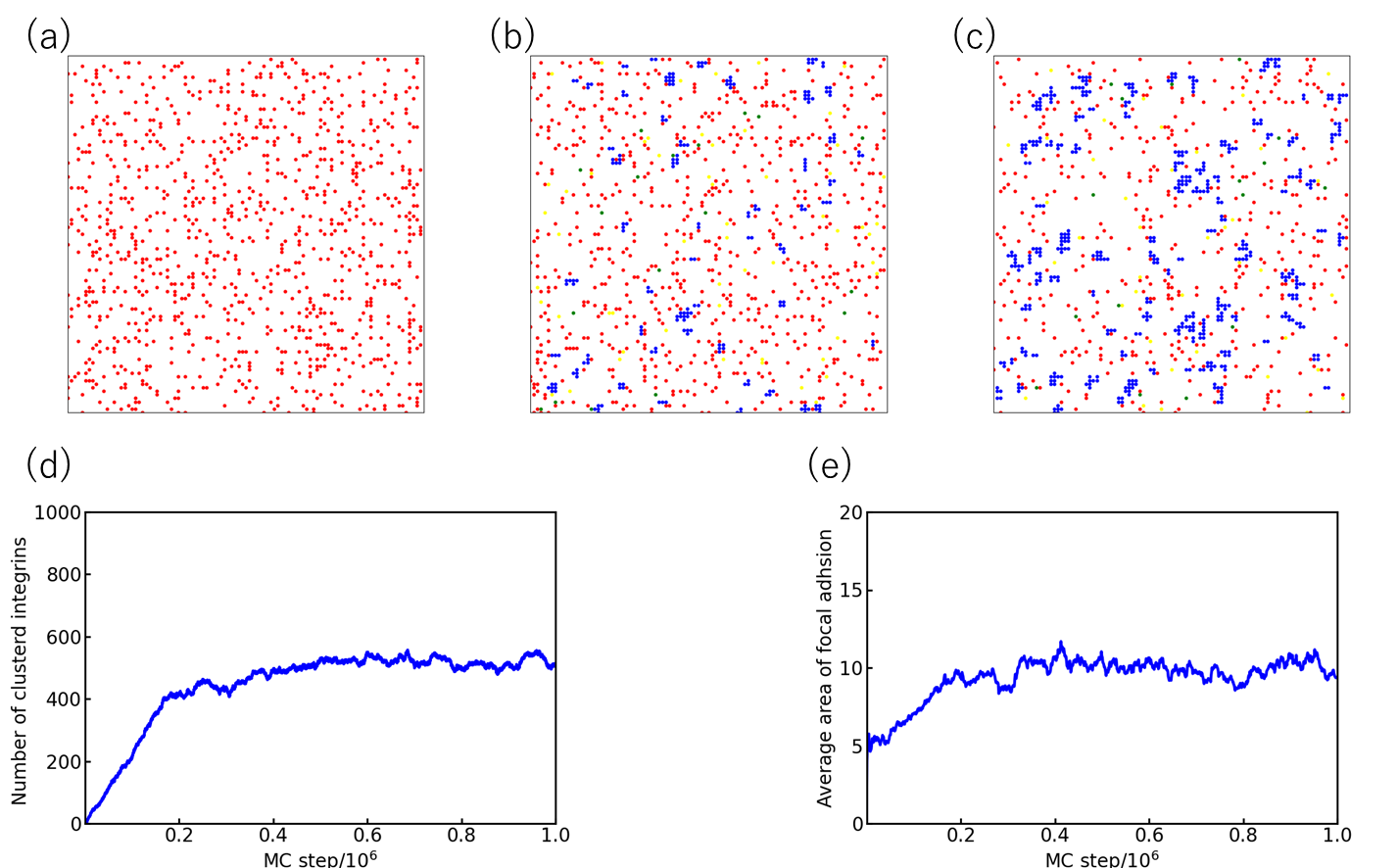
**

**Fig. S19. Variations in integrin clustering with simulation time for nanoporous Au with the pore size of 100 nm where the binding energy and the association energy depend on the pore size, *α* = 2.5 and *β* = 2.0.** (a) MS step = 0. (b) MS step = 100000. (c) MS step = 800000. Red points show non-clustered integrins and blue points show clustered integrins. (d) Variation in number of clustered integrins. (e) Variation in average area of focal adhesion

**
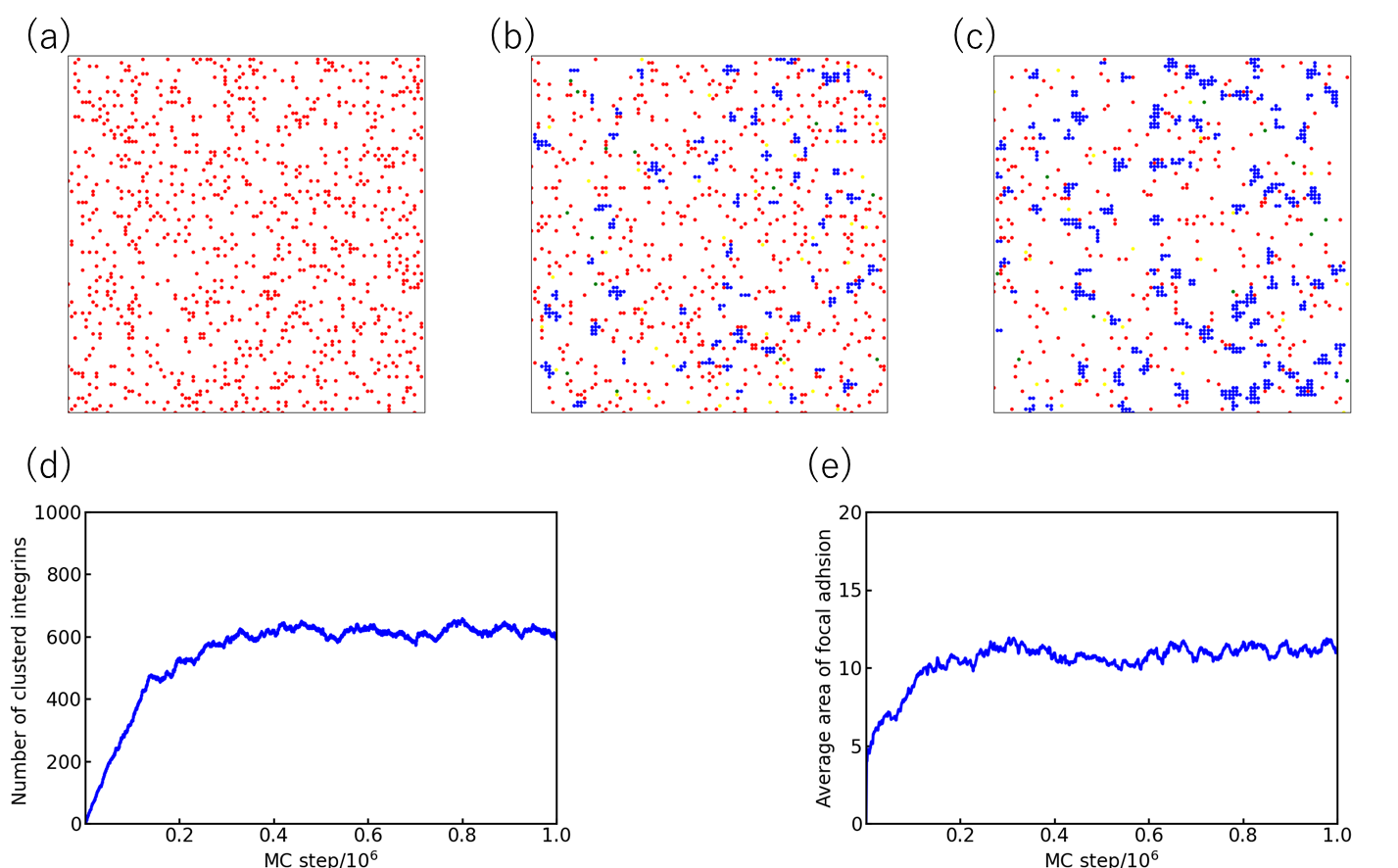
**

**Fig. S20. Variations in integrin clustering with simulation time for nanoporous Au with the pore size of 150 nm where the binding energy and the association energy depend on the pore size, *α* = 2.5 and *β* = 2.0.** (a) MS step = 0. (b) MS step = 100000. (c) MS step = 800000. Red points show non-clustered integrins and blue points show clustered integrins. (d) Variation in number of clustered integrins. (e) Variation in average area of focal adhesion

**
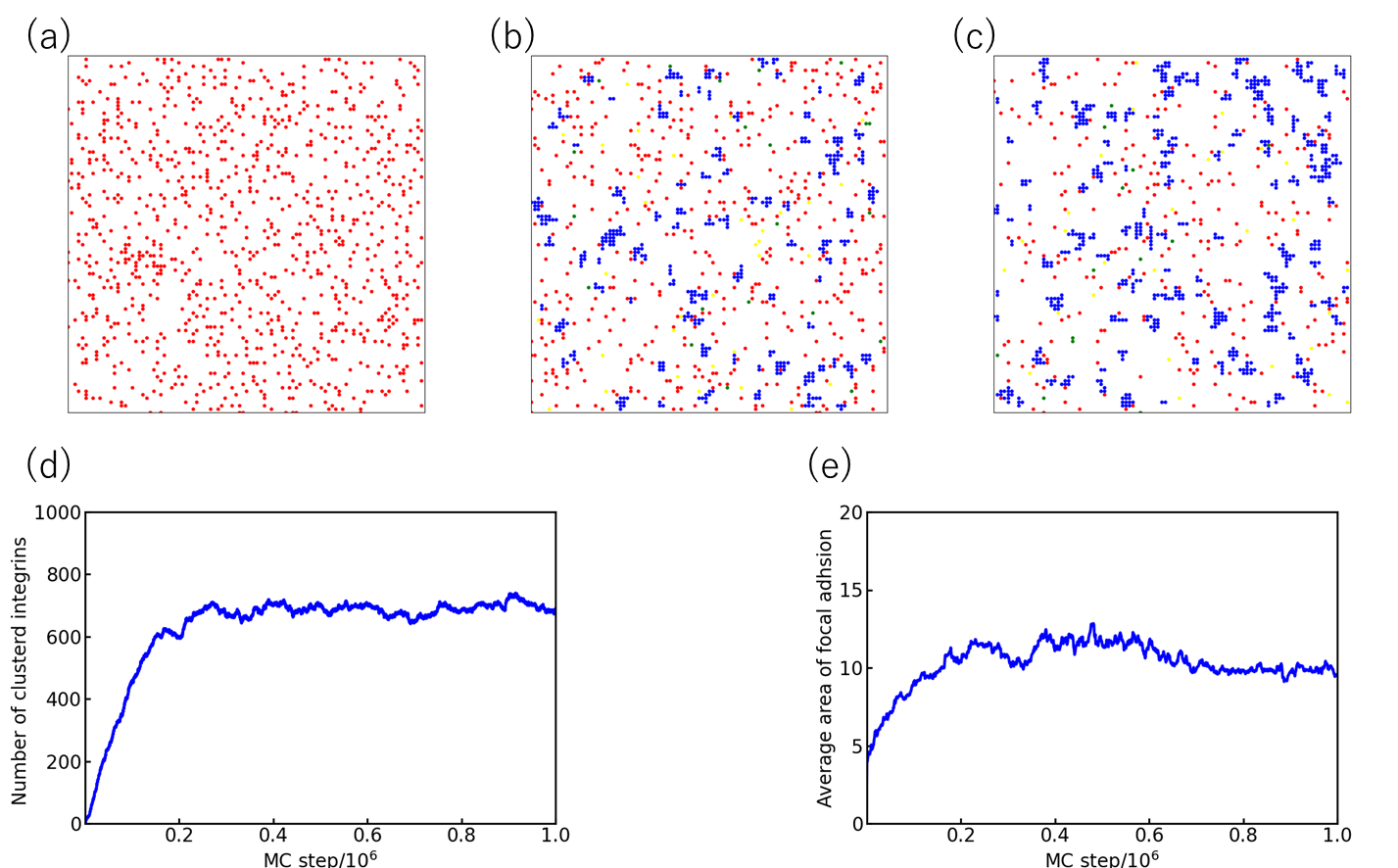
**

**Fig. S21. Variations in integrin clustering with simulation time for flat Au where** $\boldsymbol{E}_{\boldsymbol{b}}\boldsymbol{=3.5}$ **and** $\boldsymbol{E}_{\boldsymbol{c}}\boldsymbol{=6.2}$**.** (a) MS step = 0. (b) MS step = 100000. (c) MS step = 800000. Red points show non-clustered integrins and blue points show clustered integrins. (d) Variation in number of clustered integrins. (e) Variation in average area of focal adhesion
